# Uterine Fluid Extracellular Vesicles Proteome Is Altered During the Estrous Cycle

**DOI:** 10.1016/j.mcpro.2023.100642

**Published:** 2023-09-09

**Authors:** Johanna Piibor, Andres Waldmann, Keerthie Dissanayake, Aneta Andronowska, Marilin Ivask, Madhusha Prasadani, Ants Kavak, Suranga Kodithuwakku, Alireza Fazeli

**Affiliations:** 1 Institute of Veterinary Medicine and Animal Sciences, Estonian University of Life Sciences, Tartu, Estonia; 2 Faculty of Veterinary Medicine, Latvia University of Life Sciences and Technologies, Jelgava, Latvia; 3 Department of Anatomy, Faculty of Medicine, University of Peradeniya, Peradeniya, Sri Lanka; 4 Institute of Animal Reproduction and Food Research, Polish Academy of Sciences, Olsztyn, Poland; 5 Department of Pathophysiology, Institute of Biomedicine and Translational Medicine, University of Tartu, Tartu, Estonia; 6 Department of Animal Sciences, Faculty of Agriculture, University of Peradeniya, Peradeniya, Sri Lanka; 7 Division of Clinical Medicine, School of Medicine & Population Health, University of Sheffield, Sheffield, United Kingdom

**Keywords:** bovine uterine fluid, extracellular vesicles, proteome, embryo culture

## Abstract

Uterine environment is tightly and finely regulated *via* various signaling pathways mediated through endocrine, exocrine, autocrine, juxtacrine, and paracrine mechanisms. *In utero* signaling processes are paramount for normal and abnormal physiology which involves cell to cell, cells to gametes, cells to embryo, and even interkingdom communications due to presence of uterine microbiota. Extracellular vesicles (EVs) in the uterine fluid (UF) and their cargo components are known to be mediators of *in utero* signaling and communications. Interestingly, the changes in UF-EV proteome during the bovine estrous cycle and the effects of these differentially enriched proteins on embryo development are yet to be fully discovered. In this study, shotgun quantitative proteomics–based mass spectrometry was employed to compare UF-EV proteomes at day 0, 7, and 16 of the estrous cycle to understand the estrous cycle–dependent dynamics. Furthermore, different phase UF-EVs were supplemented in embryo cultures to evaluate their impact on embryo development. One hundred fifty-nine UF-EV proteins were differentially enriched at different time points indicating the UF-EV proteome is cycle-dependent. Overall, many identified pathways are important for normal uterine functions, early embryo development, and its nutritional needs, such as antioxidant activity, cell morphology and cycle, cellular homeostasis, cell adhesion, and carbohydrate metabolic process. Furthermore, the luteal phase UF-EVs supplementation increased *in vitro* blastocyst rates from 25.0 ± 5.9% to 41.0 ± 4.0% (*p* ≤ 0.05). Our findings highlight the importance of bovine UF-EV in uterine communications throughout the estrous cycle. Interestingly, comparison of hormone-synchronized EV proteomes to natural cycle UF-EVs indicated shift of signaling. Finally, UF-EVs can be used to improve embryo production *in vitro*.

## Highlights

• Evaluation of UF-EV proteome changes at day 0, 7, and 16 of the simulated bovine estrous cycle with LC-MS/MS.
• UF-EV proteome changes in key mechanisms needed for endometrial development.
• Five putative human receptivity markers (ALPL, ANXA1, B2M, OLFM4, PPIA) were significantly enriched at day 16 of the estrous cycle.
• Appropriate concentration of UF-EVs in embryo coculture is established.
• Supplementing UF-EVs to embryo culture improved blastocyst rates significantly when cocultured with follicular phase UF-EVs.

## Graphical Abstract

**Figure:**
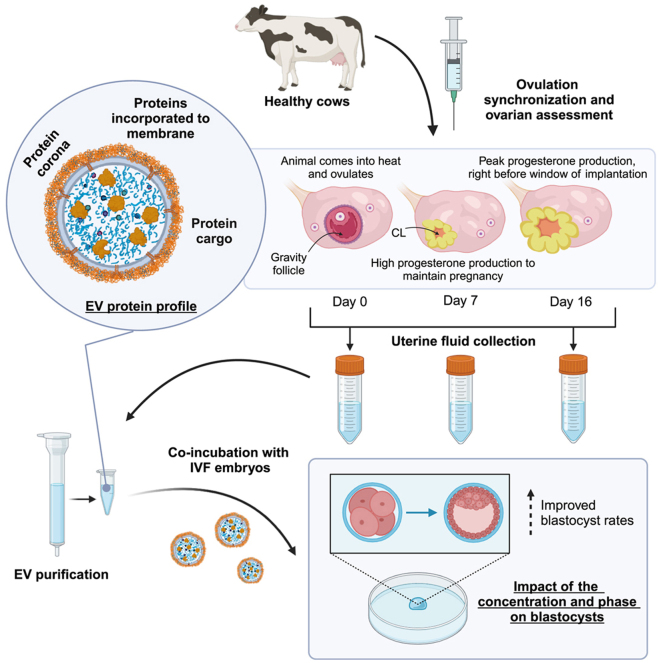

## In Brief

Piibor *et al*. investigates the uterine fluid extracellular vesicles (UF-EVs) proteome changes in different timepoints of the bovine estrous cycle and evaluates the impact of the follicular and luteal phase UF-EVs impact to in vitro embryo development.

Bovine (*Bos taurus*) estrous cycle is a dynamic process under ovarian hormone regulation that controls cows’ endometrial physiology and subsequent pregnancy status. The duration of estrous cycle in cattle is 18 to 24 days, which can be divided to two discrete periods: luteal (14–18 days) and follicular phases (4–6 days). The luteal phase starts after the ovulation when the *corpus luteum* (CL) is formed, while follicular phase begins from the regression of functional CL and ends at ovulation (1). Throughout this process, spatiotemporal changes transpire in the uterus regulated by the two key steroid hormones, progesterone and estrogen (2, 3). Uterine fluid (UF) is a mixture of growth factors, hormones, enzymes, lipids, glucose, transport proteins, and other molecules, which is typically secreted by uterine glands, epithelial cells, vasculature, and the composition of UF is known to change along with cycle dynamics (3, 4).

Uterine environment is rich in intercellular and intracellular communications owing to the complex physiology. Recent studies have discovered that these communications, especially the intercellular communications, are not only mediated by different secretory factors in UF (5) but also by the extracellular vesicles (EVs). EVs are lipid bilayer–bound nanoparticles, which contain wide range of biomolecules (*e.g.*, proteins, lipids, miRNAs, DNA) (6, 7). Cells secrete and uptake EVs from different types of biofluids such as uterine (8, 9), oviductal (8, 10), and follicular fluid (11). Depending on the cargo content, EVs influence normal physiological and pathological conditions (7). Recent studies have shown that UF-EVs modulate reproductive events such as endometrial development towards receptivity (12, 13, 14), endometrial-embryo crosstalk during pre-implantation period to support conceptus elongation and survival (15), regulation of maternal immune system allowing embryo attachment (16), and facilitation of embryo implantation (17, 18). However, the exact type of EVs and EV-cargo composition which support these biological processes are yet to be discovered.

One of the important components of EVs are proteins, which can be used as biomarkers since they reflect various states of the cells at real time in specific conditions (19). Therefore, the abrupt changes in UF-EV protein cargo may easily reflect either an optimal or suboptimal condition in the uterus for pregnancy. Some studies have been published to establish the landscape of protein changes for human UF-EVs during different time points of menstrual cycle (20) and for embryo implantation (21), but bovine UF-EV proteomic dynamic changes during the estrous cycle and how it affects endometrial development are poorly understood. However, these human studies showed that dynamic changes in EV-related proteins are needed for endometrial development and eventual embryo implantation (20, 21), for example, proteins related to cell extracellular matrix remodeling and organization (*e.g.* tenascin, alpha-2-macroglobulin, cartilage oligomeric matrix protein) (21), antioxidant activity (*e.g.* superoxide dismutase 1, glutathione S-transferase omega 1, myeloperoxidase, catalase) (20), or cell adhesion and communication (*e.g.* integrins (ITAG), mucins, agrin, moesin, transglutaminase 2, cluster determinant (CD) 47, collagen VI chains) (21).

During the preimplantation period, the bovine embryo development is not directly dependent on the uterine blood supply but solely on histotroph, a complex fluid secreted by the endometrium (3). Most bovine embryos do not reach to the implantation stage due to early embryonic losses. Overall embryonic loss in dairy cattle is around 40% from which about 2/3 occur before day 16 after fertilization (3, 22). Therefore, part of the elevated embryonic mortality during the preimplantation period can be implicated to functional incapacity of the uterine microenvironment to properly support the conceptus survival and elongation until the embryo implants to the endometrium (3). Improved understanding of the optimum uterine lumen milieu for embryo development would help to advance preventive measures to avoid early embryo losses in embryo transfer (23, 24, 25).

Embryo-maternal communication and signaling between endometrial cells during pre-implantation period is crucial in mediating embryo and endometrial growth, which both is needed for successful pregnancy. There are plethora of evidence supporting the involvement of EVs from either side in maternal-embryo cross talks in establishment of pregnancy and development of the embryo beyond the implantation (14, 24, 26, 27). However, the optimal bovine UF-EV protein composition leading to successful pregnancy is yet to be established. Therefore, the aim of the current study was to investigate the UF-EV proteomic changes in different timepoints of the bovine estrous cycle and to evaluate the impact of the follicular and luteal phase UF-EVs in promoting *in vitro* embryo development.

## Experimental Procedures

All animal experiments were approved by the Committee for Conducting Animal Experiments at the Ministry of Rural Affairs, Estonia (Approval number 200 from 9.07.2021).

### Selection of Cows for Synchronization and Collection of UF

#### Selection of Cows

Multiparous Holstein cows (*B. taurus*) aged between 3 to 6 years old were clinically examined on day 0, 6, 8, and 10 postpartum and at the start of ovulation synchronization protocol between days 26 to 30 postpartum. Cows were deemed healthy when they calved independently, did not have twins, had normal body condition (3.0–3.5 body condition score on scale of 1.0–5.0) (28), did not suffer clinical hypocalcaemia, retained placenta, metritis, strong lameness, clinical endomiketritis, or any other clinical disease diagnosed by the farm personnel during the evaluation period. Out of 20 clinically evaluated cows, nine animals met the required health criteria and were subjected to the ovulation synchronization. From the collected UF samples, six cow samples were used in this study based on the uterine cytological evaluation and assessment of the response to hormonal synchronization described below.

#### Ovulation Synchronization

Ovulation of the experimental animals was synchronized using Double-Ovsynch with additional prostaglandin F_2α_ (PGF_2α_) injection 24 h after first PGF_2α_, which has been previously shown to improve hormonal response rates (29, 30). The protocol included gonadotropin releasing hormone analog (lecirelin acetate 25 μg/ml (Dalmarelin, Fatro S.p.A.)) 2 ml per injection IM and PGF_2α_ analog (dinoprost 5 mg/ml (Dinolytic, Zoetis Belgium SA, Louvain-la-Neuve) 5 ml per injection IM. UF sample collection started 16 h after the end of ovulation synchronization protocol.

#### Cytological Evaluation of Uterine Health Status

The cytological evaluation of uteri was performed 5 days before the end of Double-Ovsynch using cytobrush technique (31). Briefly, endometrial brush (Uterobrush; Medscand Medical) was assembled to the stainless-steel device (https://worldwide.espacenet.com/publicationDetails/originalDocument?FT=D&date=20151202&DB=EPODOC&locale=en_EP&CC=EP&NR=2029026B1&KC=B1&ND=4) and autoclaved. Before sample collection, the brush was pulled inside the instrument, which was then gently inserted into vagina and guided through cervical canal to uterus. When the instrument reached the desired place in the uterus, the brush was exposed. The uterine cytology sample was collected at the base of the previously gravid uterine horn by gently rolling the brush on the endometrium. Finally, the brush was retracted back to the instrument and removed from uterus.

The cytology slides were prepared, stained, and counted according to Valdmann *et al.* (31). Briefly, two cytological examination slides were prepared by rolling the cytobrush with cells on glass slides. Slides were immediately fixed in a current of warm air using a hair dryer. Staining procedure followed May-Grünwald-Giemsa staining protocol, where first the slides were placed in May-Grünwald stain (VWR Prolabo Chemicals) for 5 min, then transferred into diluted Giemsa stain (VWR Prolabo Chemicals) for 25 min, and finally washed with distilled water. Slides were visualized under a light microscope (Olympus BX51; Olympus) using magnifications of 400× and 1000×. For each stained slide, a total of 100 epithelial cells or polymorphonuclear neutrophils (PMNs) were counted under 1000× magnification. The average percentage of PMNs out of all cells counted of the two slides had to be less than 1.0% to be included in further investigations.

#### Assessment of the Response to Hormonal Synchronization

Before the collection of UF, the uterus and ovarian structures were evaluated with transrectal ultrasonography (US) to assess the response to hormonal synchronization and detect possible abnormalities in uterus or ovaries (supplemental File S1). One cow with detected follicular or luteal cysts was excluded from the study. At sample collection day 0, US showed a uterus containing clear fluid, ovaries did not contain any CL, and there was either a presence of around 2.0 cm sized follicle or only small (up to 1.0 cm) follicle. During US examination at day 7, the uterus did not contain any fluid and the ovaries contained a CL in size range between 2.6 and 4.0 cm, follicle between 1.8 and 2.0 cm, and several small follicles up to 0.5 cm. On the 16 days of US examination, uterus did not contain any fluid, and ovaries had a CL size ranged between 2.6 and 4.0 cm, a follicle sized 1.3 to 2.5 cm with several small sized up to 0.5 cm follicles.

#### Uterine Fluid Collection

UF samples used in this study were collected from six cows on each timepoint at day 0, 7, and 16 of the estrous cycle (n = 18) after the ovulation synchronization and US assessment of uterus and ovaries as described above. From each cow, UF was collected from both uterine horns and pooled. Briefly, UF samples were acquired from cows under low sacral epidural anesthesia using xylazine (0.05 mg/kg, xylapan, Vetoquinol Biowet Sp z o.o) diluted in 5 ml of saline. Uterine horns of cows were separately flushed with 50 ml of PBS (Dulbecco’s Phosphate Buffered Saline, Sigma-Aldrich) using Foley embryo transfer catheter CH18 (Minitüb GmbH). The pooled UF (68.7 ± 15.1 ml) was collected into a plastic tube as much as possible and transported on ice for processing within 2 to 3 h of collection.

#### Sample Preparation and Storage

Differential centrifugation was performed to remove cells, cell debris, apoptotic bodies, and other impurities from collected UF samples. Following which centrifugation steps were carried out, where after each step, supernatant was transferred to another fresh tube: 250 g for 5 min at 4 °C to remove cells, 2000*g* for 10 min at 4 °C to remove cell debris, and 10,000*g* for 30 min at 4 °C to remove apoptotic bodies. After final centrifugation step, the supernatants were stored at −80 °C until EVs enrichment.

### UF-EVs Enrichment

UF samples were concentrated using Amicon Ultra 15 ml centrifugal filters (10 kDa cut-off, Merck Millipore Ltd); each sample was filtered and concentrated at 4 °C using 4000 g until final sample concentration of 500 μl.

EV isolation was performed using size-exclusion chromatography (SEC) method from the purified and concentrated UF samples (32). In brief, SEC columns were prepared by packing Econo-Pac Chromatography columns (cat. 7321010, Bio-Rad) with SEC resin (Sepharose 4 fast flow, Cytiva). Next, these vertically positioned SEC columns were washed by running through ultrapure Milli-Q water (machine type: 08.2205, TKA Wasseraufbereitungssysteme GmbH) and equilibrated with PBS. Thereafter, 500 μl of sample was added on top of the filter in the column. Immediately after adding the sample, 500 μl fractions were collected from which fraction 6 to 9 contained EVs (13). Fractions 6 to 9 were pooled and further concentrated up to 500 μl using Amicon Ultra-2 ml centrifugal filters (10 kDa cut-off, Merck Millipore Ltd) by centrifuging at 4 °C at 2000 g. Enriched EV samples were stored at −80 °C in protein low-binding tubes.

### Characterization of UF-EVs

The characterization of UF-EVs were performed on purified EV samples using nanoparticle tracking analysis (NTA) to measure particle concentration and size and transmission electron microscopy (TEM) to visualize the morphology of EVs. One sample before and after purification of EVs were submitted to shotgun proteomic analysis using liquid chromatography/mass spectrometry-mass spectrometry (LC/MS-MS) to identify the abundance of some EV-related proteins following the International Society for Extracellular Vesicles guidelines (33).

#### Nanoparticle Tracking Analysis

The number and size of particles were measured from EV samples in each cow using ZetaView (PMX 110 V3.0 instrument by Particle Metrix GmbH, Inning am Ammersee) coupled with ZetaView NTA software for data analysis. The operating instructions of the manufacturer were followed accordingly. For the auto-alignment of the instrument, 100 nm polystyrene particle size standards (cat. 10100, Applied Microspheres B.V.) were used. Samples were measured in scatter mode using camera sensitivity at 85, shutter speed at 70, frame rate at 30 fps, number of cycles at 3, and number of frames at 11 (34).

#### Transmission Electron Microscopy

The TEM analysis was performed on pooled EV samples per the estrous cycle day (day 0, 7, and 16). Briefly, formvar/carbon-coated 200 mesh grids (Agar Scientific Ltd) were placed on 20 μl droplets of purified UF-EVs from each pooled estrous cycle day for 20 min and the droplets were allowed to be absorbed on the grid. Then, the grids were incubated with 2% uranyl acetate (Polysciences) for 5 min and air-dried for obtaining contrasted images of EVs. The EVs were visualized using JEM 1400 TEM (JEOL Ltd, with Morada TEM CCD camera, Olympus) at 80 kV. Finally, the digital images of EVs were captured using a numeric camera (Morada TEM CCD camera, Olympus).

#### Mass Spectrometry for Confirmation of EV Enrichment

One UF sample before and UF-EVs after enrichment were subjected to mass spectrometry. The sample preparation and analysis with LC-MS/MS is described.

According to the International Society for Extracellular Vesicles guidelines, exosome/EV markers were picked from the dataset for identifying EV-related proteins that was either enriched or depleted after EV isolation (http://exocarta.org/exosome_markers_new) (accessed November 07, 2022). The comparison of log-transformed label-free quantification (LFQ) values of protein abundance measured from samples before and after EV isolation was performed. The data was visualized in R (v4.1.0) using package ggplot2.

### UF-EVs Proteomic Profile Analysis

#### Sample Preparation for LC-MS/MS

Proteins of the samples were precipitated using trichloroacetic acid and sodium deoxycholate protocol. Briefly, precipitation solution (sodium deoxycholate 4 mg/ml in 100% trichloroacetic acid) to 20% (v/v) was added to the samples, incubated overnight at 4 °C, and then centrifuged at 17,000 g for 15 min at room temperature. Next, the supernatant was discarded and pellet was washed with 100% acetone by incubating for 10 min and then centrifugating for 15 min at 17,000 g at room temperature. The washing process was repeated after discarding the supernatant. Rough protein concentrations were estimated by Micro BCA assay (Thermo Fisher Scientific). Precipitated proteins were then solubilized in 7 M urea, 2 M thiourea, 100 mM ammonium bicarbonate buffer, reduced with 5 mM DTT for 1 h, and alkylated with 20 mM chloroacetamide in the dark for 1 h. Predigestion was performed with 1:50 (enzyme to protein ratio) Lys-C (Fujifilm Wako Pure Chemical) for 4 h at 25 °C. Next, the solution was diluted five times with 100 mM ABC and further digested with 1:50 dimethylated trypsin (Sigma-Aldrich) for overnight at 25 °C. Samples were then acidified with TFA to 1.0% and desalted on in-house made C18 material (3M) solid phase extraction tips. Purified peptides were reconstituted in 0.5% TFA for nano-LC/MS/MS.

#### LC-MS/MS Analysis

For determining final peptide injection amounts, a twenty-fold dilution of the final sample was first pre-analyzed with LC-MS/MS, integrating the peptide signal from each sample and then equal amount of each sample was injected for a final run. LC-MS/MS analysis was performed by loading injected peptides to a 0.3 × 5 mm trap-column (5 μm C18 particles; Dionex) using an Ultimate 3000 RSLCnano system (Dionex). Peptides were eluted to an in-house packed (3 μm C18 particles; Dr Maisch) analytical 50 cm × 75 μm emitter-column (New Objective) and separated with an A to B 8 to 40% 1 h gradient (buffer A: 0.1% formic acid, buffer B: 80% acetonitrile +0.1% formic acid). Separated peptides were on-line electrosprayed to a Q Exactive HF (Thermo Fisher Scientific) mass spectrometer *via* a nano-electrospray source (positive mode, spray voltage of 2.5 kV). The MS was operated with a top-12 data-dependent acquisition strategy. Briefly, one 350 to 1400 m/z full MS scan at a resolution setting of R = 60,000 at 200 m/z was followed by higher-energy collisional dissociation fragmentation (normalized collision energy of 26) of the 12 most intense ions (z: +2 to +6) at R = 30,000. MS and MS/MS ion target values were 3,000,000 and 100,000 ions with 50 and 45 ms injection times, respectively. MS/MS isolation was carried out with 1.2 m/z isolation windows. Dynamic exclusion was limited to 20 s.

#### Data Analysis

Raw mass spectrometry data were processed with the MaxQuant software package (version 2.0.3.0). The variable modifications were set for methionine oxidation and protein N-terminal acetylation, while cysteine carbamidomethylation was defined as a fixed modification. Search was performed against UniProt (www.uniprot.org) *Bos taurus* proteome database (downloaded: 2022 June) using the tryptic digestion rule (cleavages after lysine and arginine without proline restriction). Only identifications with at least 1 peptide ≥7 amino acids long (with up to two missed cleavages) were accepted, and transfer of identifications between runs based on accurate mass and retention time was enabled. Label-free normalization with MaxQuant LFQ algorithm was also applied. LFQ ratio count (*i.e.* number of quantified peptides for reporting a protein intensity) was set to 2. Peptide-spectrum match and protein false discovery rate was kept below 1% using a target-decoy approach. All other parameters were used in their default settings.

The processed data with MaxQuant was further subjected to analysis with LFQ-analyst platform (35) to analyze and visualize proteomic differences between the bovine UF-EVs acquired at different timepoints of the estrous cycle. The data was imputed using k-nearest neighbor method and false discovery rate corrected according to Benjamini Hochberg. The cut-off values used were *P*-adjusted value = 0.05 and log2 = 1.5.

### Western Blotting

#### Sample Preparation

Western Blot (WB) analysis was performed on UF-EVs isolated from four cows at day 0, 7, and 16 of the estrous cycle (two cows were different than used in the LC/MS-MS analysis). For lysis and extraction of proteins, RIPA buffer (Thermo Fisher Scientific) with protease inhibitor cocktail (cat. 535140, EMD Millipore Corp) was used. Briefly, the buffer solution was used in 1:1 ratio with UF-EVs, mixed thoroughly, and incubated on ice for 15 min. Afterward, the samples were centrifuged at 15,000 g for 5 min at 4 °C and supernatant was collected for evaluation of protein content. The evaluation of protein content in the samples was performed using Pierce BCA Protein Assay Kit (cat. 23250, Thermo Fisher Scientific) according to manufacturer’s instructions. The absorbance of standards and the samples were measured with spectrophotometer (Ledetect 96 Microplate Reader, Biomed Dr Wieser GmbH) at 540 nm wavelength. The protein concentrations of each UF-EV sample were calculated and normalized to total protein content. Finally, the samples were denatured at 95 °C for 5 min in loading buffer with β-mercaptoethanol and equal volumes were loaded to the wells.

#### Gel Electrophoresis and Blotting

The separation of proteins was performed in a 12% SDS-PAGE. The separated proteins were transferred to polyvinylidene difluoride membranes and then blocked with 5% nonfat milk for 1 h at room temperature and incubated overnight at 4 °C with the primary antibodies. The antibodies used were Ras homolog family member A (RhoA) (cat. 67B9 #2117, Cell Signaling Technology) and β-actin (cat. 20536-1-AP, Proteintech Group Inc), in the concentrations of 1:1000 and 1:5000, respectively. The washed membranes were incubated with HRP-conjugated goat anti-Rabbit IgG secondary antibody for 1 h (1:10,000, cat. G-21234, Thermo Fisher Scientific) and after washing, bands were detected by enhanced chemiluminescence (Amersham Pharmacia Biotech) and imaged using ImageQuant RT ECL machine coupled with IQuantaCapture software (GE Healthcare Bio-Sciences AB). Band intensities were quantified using ImageJ software (National Institutes of Health and Laboratory Optical and Computational Instrumentation, https://imagej.net/ij/).

### *In vitro* Production of Embryos

#### *In vitro* Maturation of Oocytes

Unless otherwise stated, all chemicals were purchased from Sigma-Aldrich/Merck. Group embryo cultures were performed using bovine (*B. taurus*) oocytes from ovaries acquired from the slaughterhouse. During the transport, ovaries were kept in prewarmed saline buffer with gentamycin sulfate at 35 to 37 °C. After arriving in the lab, the ovaries were washed twice in prewarmed 0.9% saline buffer at 35 to 37 ºC and kept in prewarmed saline until the cumulus-oocyte complexes (COCs) were aspirated using 18-gauge needle connected to a vacuum aspiration pump (Minitüb GmbH) from the follicles between 2 to 8 mm in size to a collection tube. After aspiration, the COCs were let to settle down to the bottom of the collection tube and the excessive follicle fluid was discarded. Next, the COCs from the tube were transferred to prewarmed wash medium (15 g/L Hepes TCM-199, 26 mM sodium bicarbonate, 500 mg/L polyvinyl alcohol, 0.7 mM L-glutamine, and 50 μg/ml gentamycin sulfate) in a 35 mm Petri dish. Only quality code 1 COCs (36) were used in the study, which were washed and placed in groups of 25 to 30 in 500 μl of IVM media (TCM-199 media supplemented with 0.8% fatty acid–free bovine serum albumin (BSA) fraction V, 100 mM pyruvate, 200 mM L-glutamine, 10 mg/ml gentamycin sulfate, 10 μg/ml epidermal growth factor, and 1500 IU/ml PG600) in 4-well plates. The isolated COCs were incubated at 38.8 °C in 6% CO_2_ for 22 to 24 h.

#### *In vitro* Fertilization of Oocytes

Frozen-thawed Holstein bull’s semen (Ziard 27481 EE13993023) were used to fertilize the matured COCs. Briefly, the frozen sperm straw was removed and thawed in 37 °C water bath for 1 min. Then the straw was cut from the ends and emptied to a tube containing prewarmed sperm wash media (supplemented with 60% lactic acid, 10 mg/ml gentamycin sulfate, and 500 μg/ml phenol red) and washed by centrifuging at 320*g* for 5 min. The washing was repeated. Washed spermatozoa were evaluated and counted under a light microscope using a hemocytometer. Sperm concentration was adjusted to the concentration of two million/ml in the IVF-TALP media (supplemented with 0.6% fatty acid–free BSA fraction V, 100 mM pyruvate, 1 mg/ml heparin, penicillamine hypotaurine epinephrine (20 mM penicillamine, 10 mM hypotaurine, 1 mM adrenaline), and 10 mg/ml gentamycin sulfate). The matured COCs were washed and placed in 500 μl IVF-TALP media in 4-well plate and in vitro fertilization was performed at 38.8 °C in 6% CO_2_ for 18 h.

#### *In vitro* Culture of Presumptive Zygotes

COCs were transferred to a prewarmed wash media. Cumulus cells were removed from presumptive zygotes by vortexing for 2 min. The denuded embryos were transferred to 500 μl SOF media (supplemented with 50 BME amino acids solution, 100× MEM non-essential amino acids solution, 100 mM pyruvate, 10 mg/ml gentamycin sulfate, 200 mM L-glutamine, and 0.8% EV-depleted fatty acid–free BSA fraction V), which was covered with 400 μl mineral oil. The presumptive zygotes were cultured at 38.8 °C in 6% CO_2_ and 6% O_2_ for 8 days. Embryos were morphologically evaluated at day 2, 5, and 8 days postfertilization, and the developmental stages were assessed as previously described (36).

#### Supplementation of UF-EVs to Embryo Culture

The embryo cultures were supplemented with the UF-EVs at day 2 postfertilization. The concentration of UF-EVs was measured with NTA before supplementing to embryo culture. Two different embryo culture supplementation experiments were carried out: 1) determining the concentration of supplemented UF-EVs needed for the highest rate of blastocysts and 2) evaluation of follicular and luteal phase UF-EVs impact on embryo culture development. In the first experiment, the impact of UF-EVs on bovine embryo morphological development to blastocysts in group embryo cultures (n = 25–30 per group) supplemented with the luteal phase UF-EV in concentrations of 4.32 × 10^9^, 4.32 × 10^8^, and 4.32 × 10^7^ particles/ml (pooled from three cows) in the media compared to control (no EV supplementation) was evaluated. Based on the results of the first experiment, 4.32 × 10^8^ particles/ml were used in all subsequent supplementation groups of pooled three different UF-EVs at follicular and luteal phases. The blastocyst rates were compared between UF-EVs supplemented cultures from different phases and non-EV culture. Both experiments were performed in six replicates.

### Experimental Design and Statistical Analysis

**Fig. 1:**
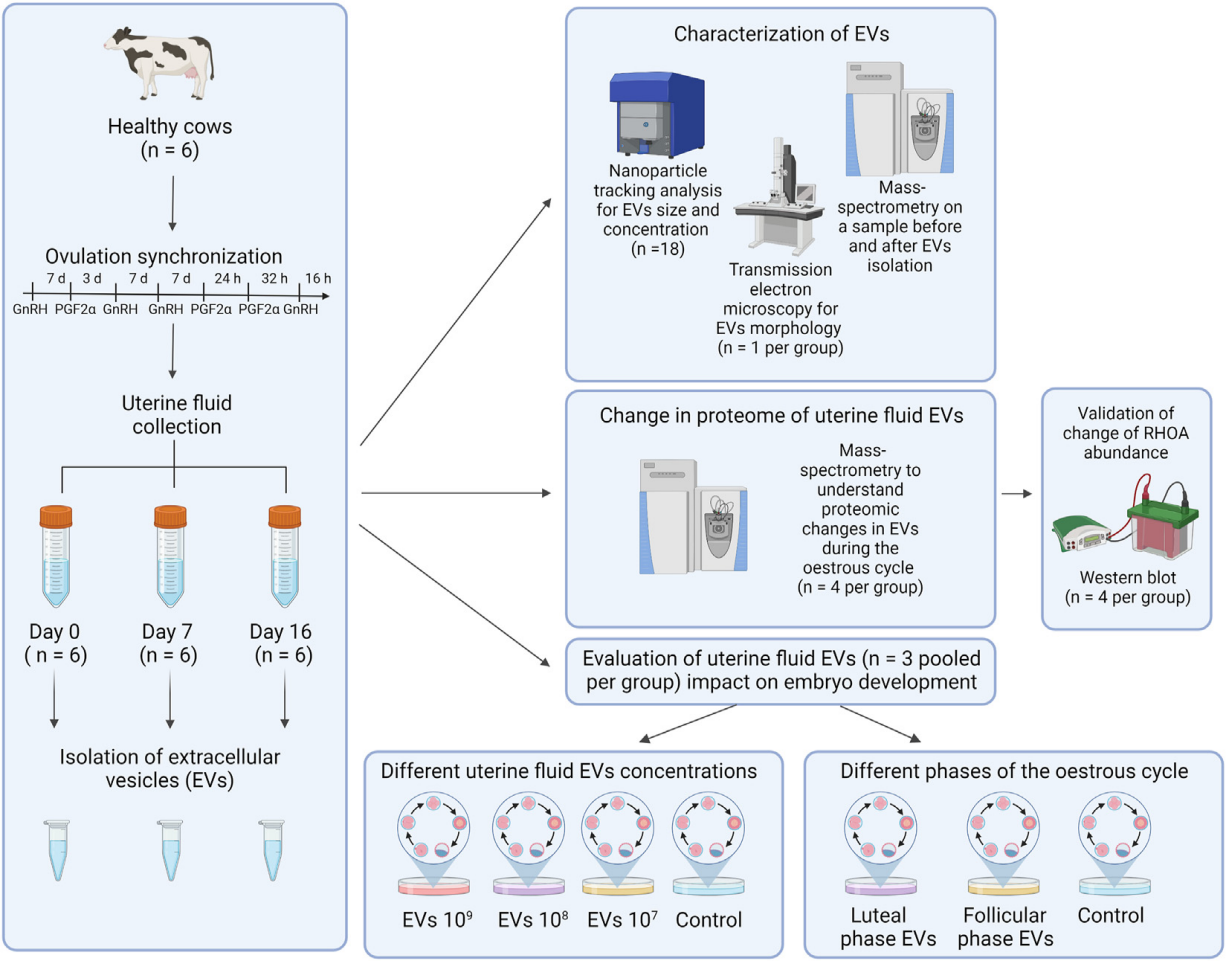
**Experimental design.** Six healthy cows were selected for the study who were subjected to modified Double-Ovsynch for synchronizing ovulation, after which uterine fluid was collected from every cow on day 0 (n = 6), 7 (n = 6), and 16 (n = 6) of the estrous cycle. From all uterine fluids (n = 18), the extracellular vesicles (EVs) were isolated using size-exclusion chromatography–based method. Uterine fluid EVs were characterized with nanoparticle tracking analysis for EVs size profile and concentration (n = 18), transmission electron microscopy for EVs morphology (n = 1 pooled per group), and mass spectrometry on a sample before and after EVs isolation to evaluate the protein enrichment of EV-related proteins after isolation. Moreover, change in the proteome of uterine fluid EVs during the different timepoints of the bovine estrous cycle was evaluated using mass spectrometry (n = 4 per group). The change of Ras homolog family member A (RHOA) protein abundance was validated using Western Blot analysis (n = 4 per group). Lastly, the uterine fluid EVs (n = 3 pooled per group) impact on embryo development was assessed. In the first experiment, influence of different concentrations (10^9^, 10^8^, and 10^7^ particles/ml) of uterine fluid EVs on blastocyst rates was evaluated and compared with a control (no EVs added to the culture). In the second experiment, influence of uterine fluid EVs at luteal or follicular phase to the blastocyst rates was assessed and compared with a control (no EVs added to the culture). The figure was created with BioRender.com.

The overall experimental design of the experiments is depicted in the Figure 1. In total, six healthy cows, which were subjected to a modified Double-Ovsynch protocol for ovulation synchronization, were used for this study. UF were collected from the cows on day 0, 7, and 16 after ovulation in the estrous cycle. The cows did not have follicular or luteal cysts identified during the ultrasonographic investigation. All collected UF samples contained <1.0% of PMNs in cytological examination. The EVs from the six cows’ UF samples (n = 18) were isolated using SEC-based methodology and characterized as described in this study. All *in vitro* experiments were conducted according to complete randomized design.

Plotting of the particle concentration profiles acquired from NTA analysis and blastocyst rates were performed using GraphPad Prism v9.3.0.463 (GraphPad Software, https://www.graphpad.com/features). Data is shown as mean ± SD. Log-rank test in R was used to compare the survival probability of cleaved embryos developing to blastocysts of different EV coculture groups and control group. The parameters which were used in the analysis were evaluations on day 3 for cleavage, day 5 for the morula stage, and day 8 for blastocyst stage postfertilization. The differences between blastocyst rates were evaluated in R using logistic regression analysis. The P-adjustment for logistic regression was performed with Tukey method. The tests were performed on the log odds ratio scale. The differences were considered statistically significant when *p* ≤ 0.05.

## Results

### Characterization of UF-EVs

**Fig. 2:**
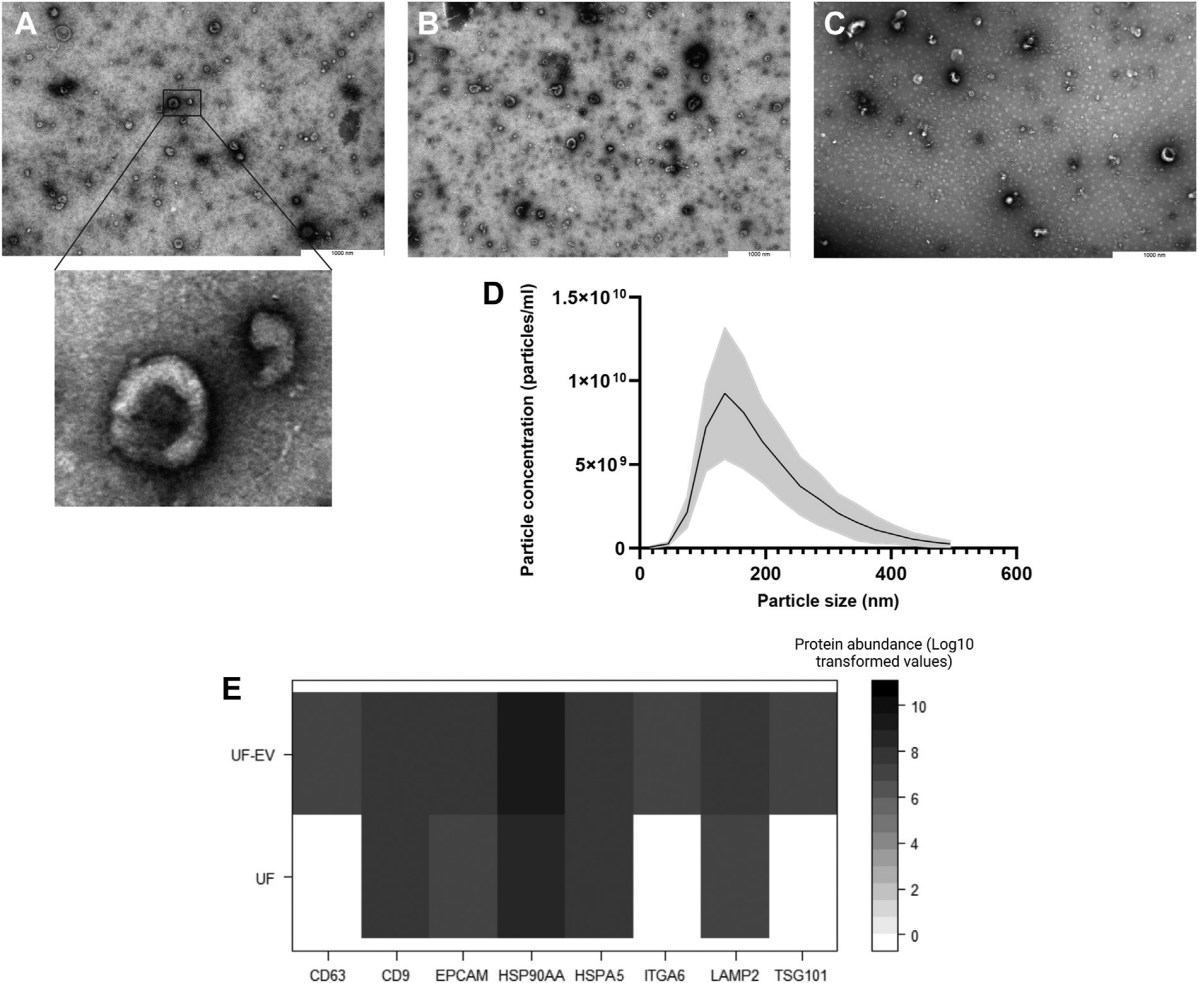
**Characterization of uterine fluid extracellular vesicles.** The characteristic cup-shaped structures were identified with transmission electron microscopy from UF-EVs samples at day 0 (*A*), day 7 (*B*), and day 16 (*C*). The particle size profile showed particles in the range of 40 to 375 nm (*D*). The SD is depicted in the figure with the color gray. Protein enrichment after UF-EVs isolation was seen in exosome-related proteins, for example (*E*): cluster determinant (CD) 63 and 9, epithelial cell adhesion molecule (EPCAM), heat shock protein (HSP) 90AA1 and A5, integrin subunit alpha 6 (ITAG6), lysosomal-associated membrane protein 2 (LAMP2), and tumor susceptibility gene 101 (TSG101). EV, extracellular vesicle; UF, uterine fluid.

Characteristic cup-shaped vesicular structures in the enriched EV samples were identified using TEM, which is a typical morphology for EVs (Fig. 2, *A*–*C*). Moreover, particle sizes were measured with NTA in the typical size range of EVs between 40 to 375 nm (Fig. 2*D*) with an average particle size of 193.7 ± 21.6 nm. LC-MS/MS analysis identified 90 EV-related proteins (supplemental File S2), which have been previously associated with exosomes (ExoCarta: Exosome Markers, 2022.). From these proteins, 42 were enriched in the UF-EVs, such as CD63, CD9, epithelial cell adhesion molecule, heat shock protein90AA, heat shock protein A5, ITAG6, lysosomal-associated membrane protein 2, and tumor susceptibility gene 101 confirming the enrichment of EVs (Fig. 2*E*).

### The Significant Differences in UF-EVs Protein Profile during the Bovine Estrous Cycle

**Fig. 3:**
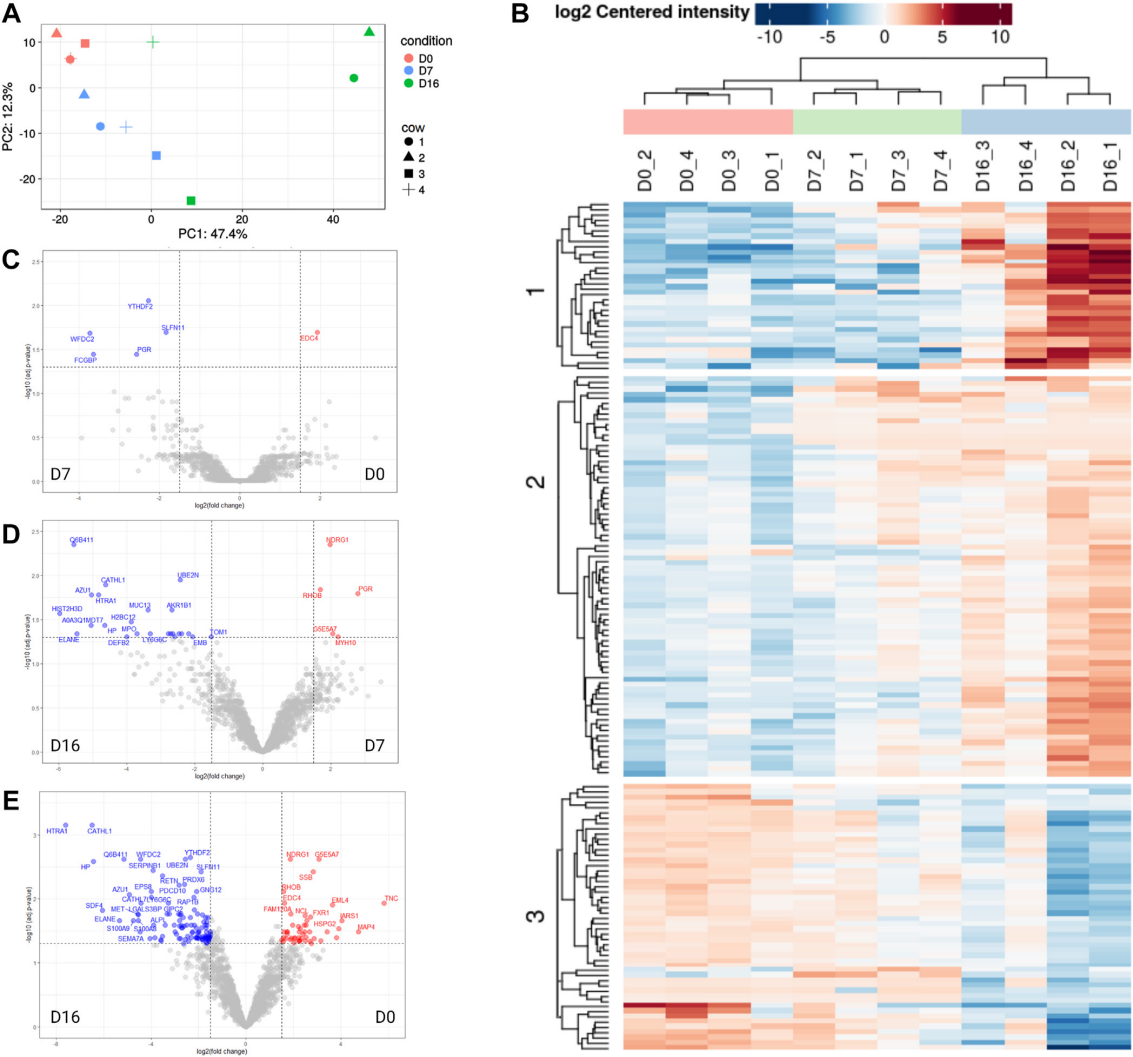
**The UF-EV protein profile dynamics between day 0, day 7, and day 16 of the bovineestrous cycle.** The principal component analysis (PCA) showed separation in overall protein enrichment patterns between D0, D7, and D16 of the estrous cycle (*A*). Heatmap showed dynamic changes of significantly different protein enrichment from D0 to D16 of the estrous cycle (*B*). Between D0 *versus* D7, there was one protein enriched and five depleted (*C*). At D7 *versus* D16 of the estrous cycle, five proteins were enriched and 24 depleted (*D*). The highest difference was between D0 and D16, where 49 proteins were enriched and 105 depleted between the timepoints (*E*). EV, extracellular vesicle; UF, uterine fluid.

The UF-EV protein enrichment between the different timepoints of the estrous cycle was significantly different (*p* ≤ 0.05) (Fig. 3). The principal component analysis showed separation in overall protein enrichment patterns between day 0, 7, and 16 of the estrous cycle (Fig. 3*A*). However, the variation of the UF-EVs protein enrichment within the groups between the cows increased towards the later timepoint of the estrous cycle (Fig. 3*A*). The heatmap analysis showed dynamic changes in enrichment of these proteins from day 0 to day 16, which formed three different clusters (Fig. 3*B*). The cluster one proteins enrichment increased towards the end of the estrous cycle from day 0 to 16 of the estrous cycle (Fig. 3*B*) and they were related to immune functions (*e.g.* LCN2, AZU1, ESPS8, CATHL1) and antioxidant activity (MPO, HP, S100A9, S100A8). Similarly, increasing protein abundance towards day 16 was observed in the cluster 2 (Fig. 3*B*), where proteins were in the pathways related to Ras signaling pathway (*e.g.* GNG5, CALM, CDC42, RAP18, RHOA) and actin cytoskeleton organization (*e.g.* ACTC1, BAIAP2, CDC42, RAC1, ANXA1). However, the protein abundance in the cluster 3 decreased from day 0 towards day 16 of the estrous cycle (Fig. 3*B*). These proteins were related to processes in gene expression (*e.g.* DHX15, ILF2, SSB, DHX9, EPRS) and different metabolic processes such as nitrogen metabolism (*e.g.* DHX15, IARS, RARS, RPL10) and negative regulation of hyaluronan biosynthetic process (AP2A1, CLTC) in the endometrium.

In total, 159 proteins were significantly different between the timepoints (supplemental File S3). At day 0 of the estrous cycle, only one protein was significantly enriched, and five proteins were significantly depleted compared to the day 7 (Fig. 3*C*). At day 7, compared to day 16 of the estrous cycle, five proteins were enriched and 24 proteins depleted, while at day 0 compared to day 16, there were 49 proteins enriched and 105 depleted (Fig. 3*E*).

### The UF-EV Proteins Involved in Biological Processes Essential for Early Embryo Development and Endometrial Receptivity

**Fig. 4:**
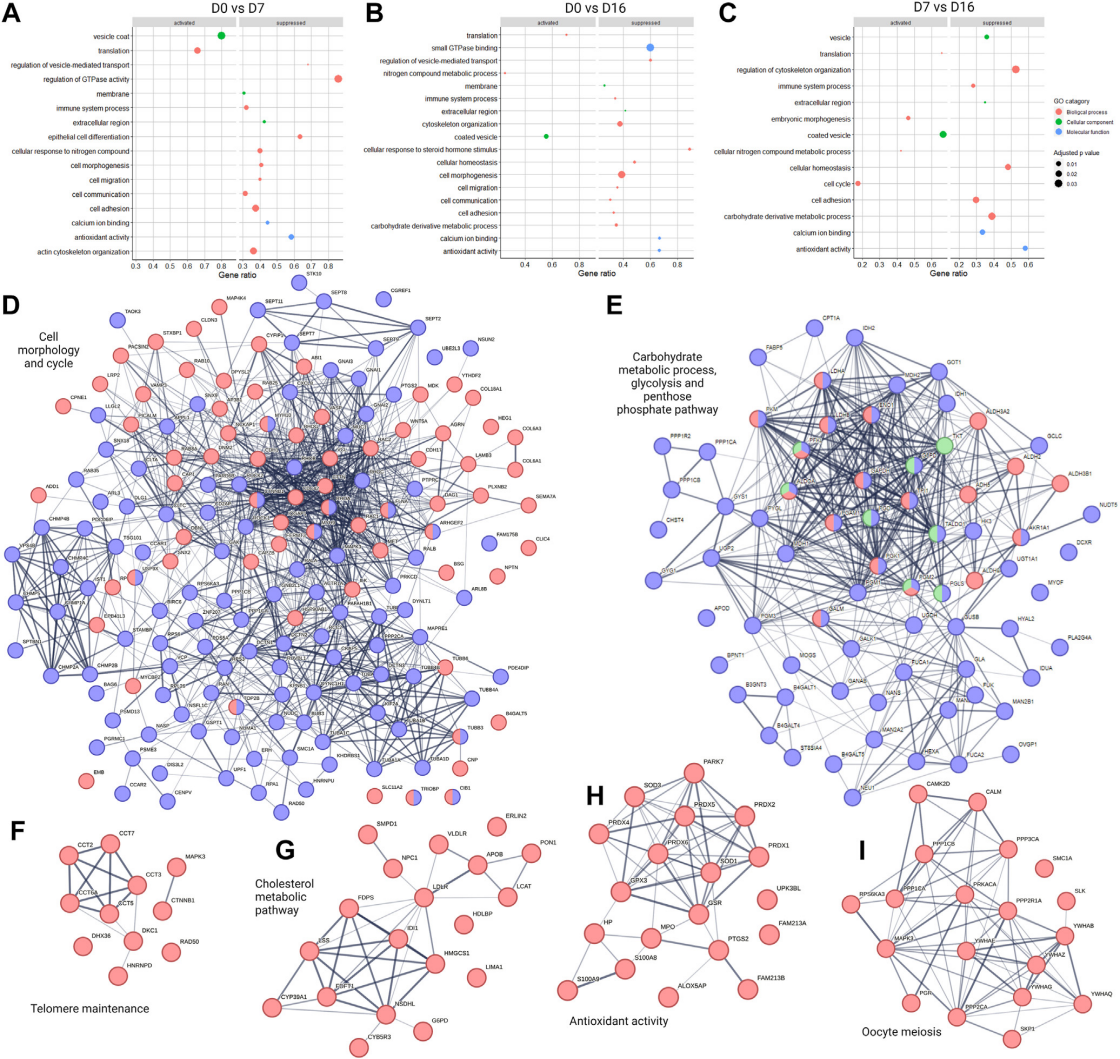
**Pathways and protein interactions of uterine fluid extracellular vesicles involved in early embryo development.** Differential enrichment of selected gene ontology (GO) pathways of biological processes, molecular function, and cellular component between day 0 (D0) and day 7 (D0) (*A*), D0 and day 16 (D16) (*B*), and D7 and D16 (*C*). The interactions between detected proteins in the cell morphology (*blue*) and cell cycle (*red*) pathways (*D*). Detected protein interactions in the carbohydrate metabolic (*blue*), glycolysis (*red*), and pentose phosphate pathway (*green*) (*E*). Pathways and their proteins interactions of telomere maintenance (*F*), cholesterol metabolic pathway (*G*), antioxidant activity (*H*), and oocyte meiosis (*I*).

The LC/MS-MS analysis identified total of 1699 proteins in the UF-EV samples. These proteins most commonly could be found in coated vesicles, extracellular region and membrane, according to gene ontology (GO) cellular component terminology (Fig. 4, *A*–*C*). Functional enrichment analysis of these proteins showed several pathways involved in regular endometrial physiology and early embryo development (Fig. 4). Several of these pathways were activated at day 7 or day 16 of the estrous cycle compared to day 0, such as cell morphogenesis, cell adhesion, antioxidant activity, and cellular homeostasis, while two GO biological process terminology, translation, and nitrogen compound metabolic pathways were suppressed (Fig. 4, *A* and *B*). On day 7 compared to day 16 of the bovine estrous cycle, proteins related to activation of embryonic morphogenesis, cell cycle, translation, and cellular nitrogen compound metabolic processes were significantly increased, while some proteins related to pathways such as cellular homeostasis, cell adhesion, carbohydrate metabolic process, and antioxidant activity were suppressed (Fig. 4*C*). The protein–protein interactions for selected pathways important for early embryo development and for healthy embryo nutritional needs during the peri-implantation period, such as cell morphology and cycle, were also detected (Fig. 4*D*). Furthermore, carbohydrate metabolic process, glycolysis and pentose phosphate pathway (Fig. 4*E*), telomere maintenance (Fig. 4*F*), cholesterol metabolic pathway (Fig. 4*G*), antioxidant activity (Fig. 4*H*), and oocyte meiosis (Fig. 4*I*)–related protein interaction networks were also identified.

**Fig. 5:**
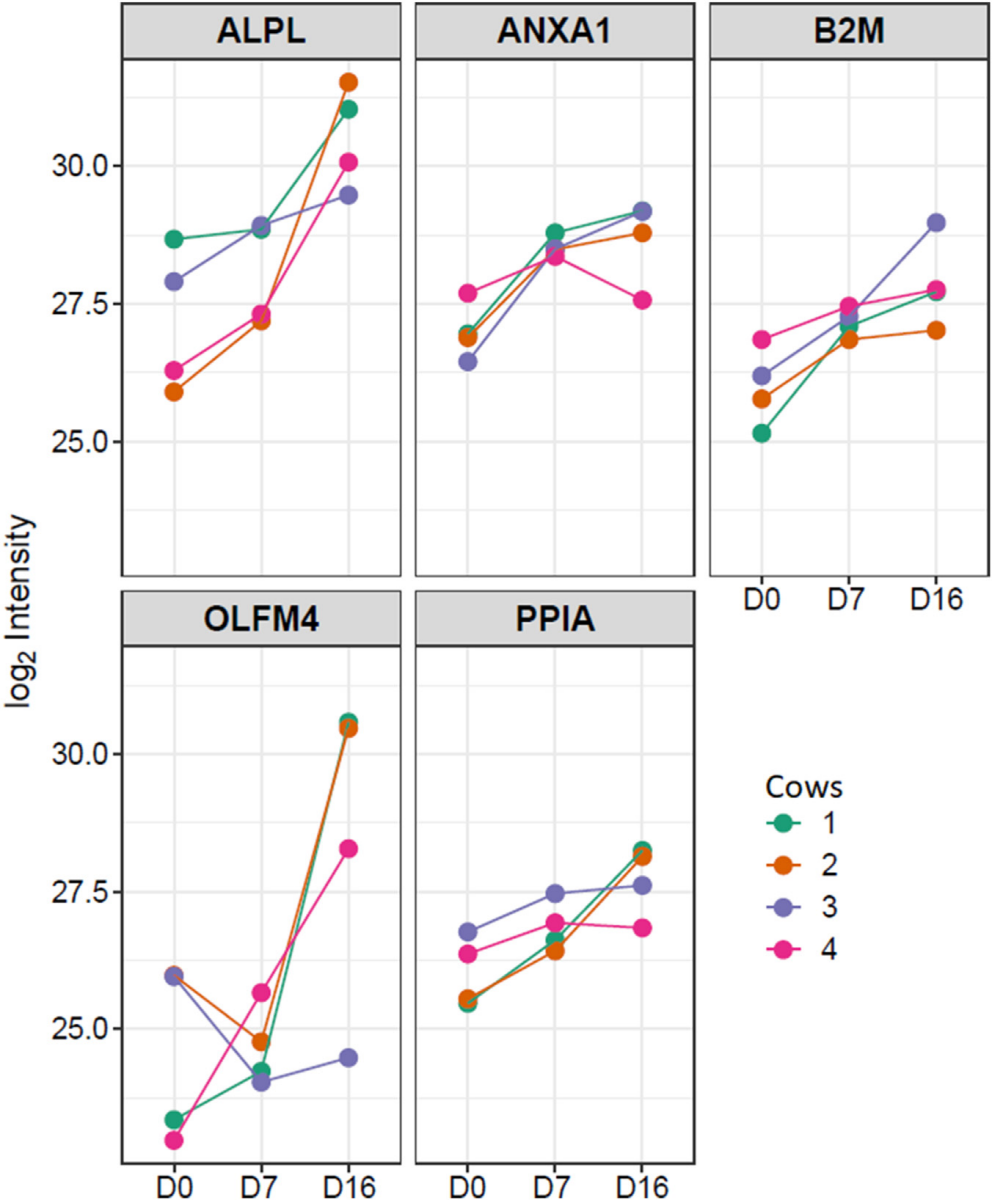
**The dynamic changes of significantly enriched receptivity-related proteins between day 0 and 16 of theestrous cycle.** The changes of protein alkaline phosphatase tissue-nonspecific isoenzyme (ALPL), Annexin A1 (ANXA1), Beta-2-microglobulin (B2M), Olfactomedin 4 (OLFM4), and peptidyl-prolyl cis-trans isomerase A (PPIA) log 2 intensities of different cows on day 0, 7, and 16 of the estrous cycle are represented in different colors.

In total, 560 UF-EV proteins were commonly measured from all cows at all the timepoints. Interestingly, out of these proteins, several have been previously identified as potential endometrial receptivity markers for humans (20, 21, 37, 38) (supplemental File S4). However, only some of them (ALPL, ANXA1, B2M, OLFM4, PPIA) were significantly enriched at day 16 (peri-implantation period) of the estrous cycle compared to day 0 and not significant between any other measured timepoints (Fig. 5).

#### Validation of LC/MS-MS Results

To validate the LC/MS-MS results, we selected RHOA, which was measured significantly higher at day 16 than day 0 of the estrous cycle, using WB analysis. The WB results showed similar results compared to LC-MS/MS analysis, therefore confirming the RHOA change in UF-EVs during the estrous cycle (supplemental File S5) indicating the trend we got from MS/MS analysis is correct.

### The Impact of UF-EVs on Embryo Development

**Fig. 6:**
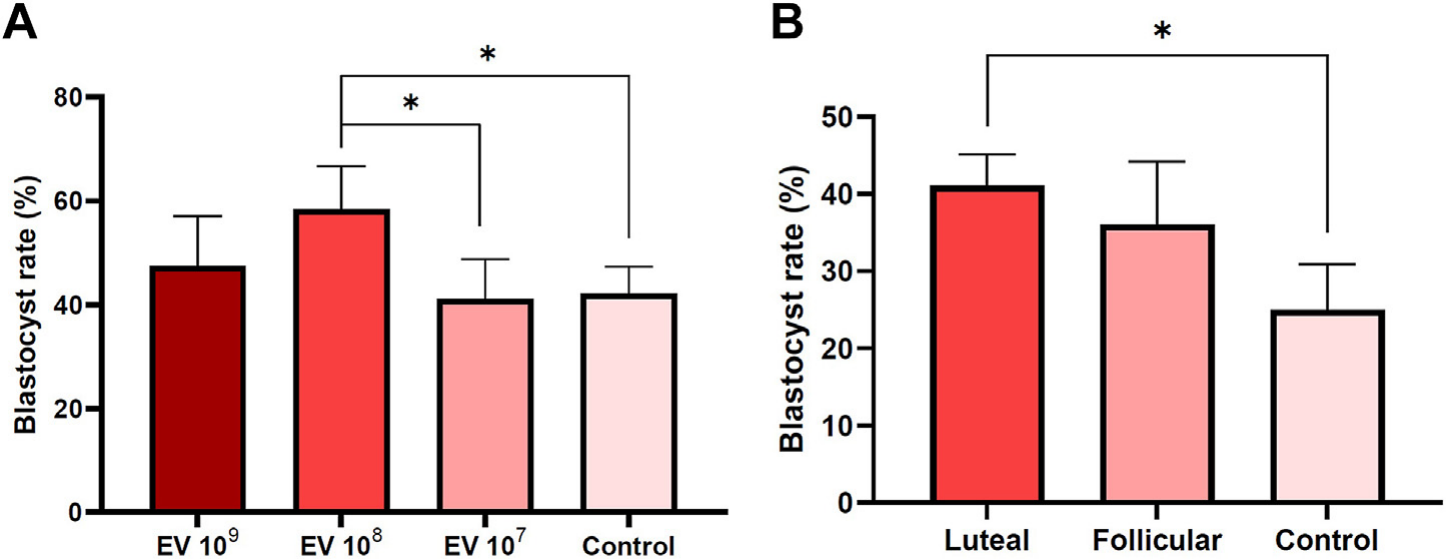
**Uterine fluid extracellular vesicles concentration andestrous cycle phase influence on embryo development.** Embryo culture was supplemented with different concentrations of UF-EVs, where the highest rate of blastocysts was achieved using UF-EVs in the concentration of 10^8^ particles/ml. The survival probability of cleaved embryos to blastocysts in UF-EVs coculture group with 10^8^ particles/ml was significantly higher between control group (*p* = 0.03) and UF-EVs coculture group with 10^7^ particles/ml (*p* = 0.04) (*A*). Embryo cultures were also supplemented with UF-EVs acquired from luteal and follicular phases of the estrous cycle, which increased the blastocyst rates compared to control. However, the probability of embryos surviving to blastocysts were significantly higher only in luteal phase UF-EV coculture group (*p* = 0.02) than the control group (*B*). EV, extracellular vesicle; UF, uterine fluid. ∗ *p* ≤ 0.05.

In the first embryo culture experiment, the influence of different concentrations of pooled bovine UF-EVs (n = 3) on blastocyst rate was evaluated. The highest blastocyst rate (58.4 ± 8.3%; confidence interval: 47.7–69.0 from cleaved embryos) was observed in the group supplemented with UF-EVs concentration of 10^8^ particles/ml (Fig. 6*A*). The survival of cleaved embryos up to blastocysts in the group supplemented with UF-EV at 10^8^ particles/ml was significantly higher than the control group where no EVs were supplemented (*p* = 0.03) and the group 10^7^ particles/ml of UF-EVs were supplemented (*p* = 0.04). However, 10^7^ and 10^9^ particles/ml had no significant effect on the embryos compared to control.

Based on the results of the first experiment, the second experiment UF-EVs supplementation was performed with UF-EVs at a concentration of 10^8^ particles/ml isolated from follicular and luteal phases. In this experiment, the phase of the estrous cycle influence to embryo development was investigated. Embryo cultures supplemented with luteal UF-EVs showed significantly (*p* = 0.03) improved blastocyst rates (41.0 ± 4.0%; confidence interval: 32.4–50.4 from cleaved embryos) compared to the control group (25.0 ± 5.9%) (Fig. 6*B*). Moreover, the follicular phase UF-EVs–supplemented group also showed increased blastocyst rates (36.0 ± 8.2%) compared to control; however, it was not statistically significant (*p* = 0.19) (Fig. 6*B*). The survival of cleaved embryos to blastocysts was significant higher (*p* = 0.02) only between luteal phase EVs supplemented and control groups.

## Discussion

The cyclical dynamic regulation of changes in UF and its components, including EVs, provide an optimal microenvironment for endometrial and embryo development, which is essential for the normal physiology and for a successful pregnancy (3, 8, 9). However, in many eutherian mammals including human and farm animals, the exact roles of UF-EVs in the normal endometrial functions and pregnancy establishment are still unknown. Therefore, in this study, we tried to delineate the possible UF-EV proteomic changes in healthy cattle at different time points of the estrous cycle and its potential in influencing endometrial physiology and bovine *in vitro* embryo development.

UF contains different biomolecules, which potentially could be co-isolated with EVs, especially in the EV protein corona. Some of these co-isolates could be protein coagulates, which are measured together with UF-EV proteins. To our knowledge, there is no EV isolation method developed which could separate purely the EVs from all other types of non-EV particles. However, our study showed enrichment of EV-related proteins in isolated samples compared to raw UF, which shows successful enrichment of EVs from UF. The measured protein content of EVs could be found inside the EVs, incorporated to the EV membrane or in the EV protein corona (7, 39). Most of the identified proteins were commonly found in coated vesicles, extracellular region, and membrane according to the GO cellular component terminology analysis. This indicates that our EV isolation methodology allowed high enrichment of functional EVs from cow UF.

In one of our previous studies, we have identified 2587 proteins in the UF-EVs enriched from cows with natural cycle, where 41 proteins were differentially expressed between follicular and luteal phases of the estrous cycle (13). When comparing the results of present study of UF-EV proteome acquired from synchronized cows and the previous UF-EV proteome study from naturally cyclic cows, we identified that 51.7% of proteins were overlapping in both datasets. However, from the differentially expressed proteins, only three proteins (TNC, OXTR, WFDC2) were common for both studies (supplemental File S6).

It is well established that that protein composition of UF is altered by hormonal stimulation in humans compared to the natural cycle (40). Interestingly, our comparison with natural cycle–obtained EVs *versus* hormonal synchronized cycle EVs proteome also indicate shift in protein enrichment. This might be due to the influence of hormonal synchronization to the immune properties of the endometrium, which can influence the endometrial functions and shift the receptivity period. The female reproductive tract and its immune response is known to be under sex hormones regulation (41). For example, increases in endometrial thickness is more rapid in induced estrous compared to naturally cyclic cows due to shorter high level hormonal changes in synchronized cows (42). Dependent on hormonal response, the expression patterns of endometrial cells may be influenced as well, in which changes are reflected in specific biomolecules needed to reach to receptive endometrium (43). Therefore, in artificial insemination, the inclusion and influence of hormonal uterine preparation for implantation should be further studied to understand its potential mechanisms to improve the bovine fertility. However, some of the differences observed in the comparison of two data sets might have been influenced by the different MS analysis methods used in the two studies, variations of sampling times in the bovine estrous cycle, differences between sampling cows with synchronized ovulation and natural cycle, and individual cow variations might also have affected the results. Nevertheless, this comparisons of natural and simulated cyclical changes of the bovine EV proteomes are the first step to understand the hormonal influence on uterine proteome. Further analysis with machine learning approach with algorithm to adjust the expression level could be suggested to minimize the mass spectrometry experimental variations (44).

The development of endometrial cells towards receptivity involves several molecular changes, which is dependent on cell-to-cell signaling, either promoting or depressing different functions (45). In the current study, we saw many pathways and their enrichment changes in different timepoints of the estrous cycle that are key mechanisms needed for endometrial development. These pathways were related to cell adhesion and morphogenesis, epithelial cell migration and differentiation, cytoskeleton organization, and immune modulation. All these pathways in normal healthy endometrium must be in balance, even though the expression patterns are constantly changing throughout the estrous cycle. Some studies have shown that abnormal activation of Ras-signaling pathway can lead to dysregulation of several signaling pathways, which would lead to eventual implantation failure (46). In our study, we noticed a dynamic increase of proteins related to Ras-signaling pathway responsible for cell proliferation and differentiation from day 0 to day 16 of the estrous cycle. One of the differentially enriched Ras family proteins was RHOA, which is involved in endometrial growth. The overexpression of RHOA has shown abnormal endometrial migration, which is shown to be blocked with interaction to ezrin (47). The local feedback loop and its balance between RHOA and ezrin is necessary for the proper morphological development of endometrium. Therefore, further studies are needed to understand the signaling interactions of UF-EV proteome components in healthy endometrium essential for different processes of endometrial development, which could lead to potential therapies for abnormal endometrial development leading to create unhealthy uterine microenvironment.

The majority of pregnancy loss in cattle occurs prior to maternal recognition of the pregnancy on day 16 (3, 22). During this timepoint, spatio-temporal changes occurring in the uterine tissue are clearly reflected in the UF-EVs (5). These changes lead to formation of a receptive endometrium that is an essential prerequisite for the establishment of pregnancy. In human assisted reproductive technology (ART) settings, many attempts have been made to define endometrial receptivity markers even using EV cargoes (17, 48); however, the endometrial receptivity markers for cattle are yet to be determined (49, 50). In our study, with the purview of comparative proteomics, we identified several bovine UF-EV proteins that were proposed to be putative receptivity markers for humans (20, 21, 37, 38). However, only five of them (ALPL, ANXA1, B2M, OLFM4, PPIA) were significantly enriched towards the day 16 of the estrous cycle. Establishing the expression patterns of receptivity-associated proteins could determine the window of implantation as well as predict the implantation success in the ART settings (20) where in cattle embryo transfer is performed in a very blind endometrial status. Thus, future studies are required to understand the molecular changes in UF, which can predict the chances of pregnancy establishment, and potentially reduce the pregnancy losses due to issues with maternal recognition. Since UF can be obtained using minimal invasive procedures, devising an on-farm base test could be achieved.

During the peri-implantation period, the growth and survival of the embryo are influenced by the composition of UF from the maternal side (3, 8, 9). In the present study, we identified many UF-EV proteins and their pathways that are essential for early embryo development. One of the important pathways for embryo development identified was antioxidant activity, where HP, MPO, S100A8, and S100A9 were enriched at day 16 compared to day 0 of the estrous cycle. Many studies have shown that antioxidants are essential to protect embryos from reactive oxygen species (ROS) damages, which cause DNA damage, delay in embryo development, and that lead to embryo death (51, 52, 53). ROS are by-products of cells’ normal oxygen metabolism, which plays roles in cellular signaling, homeostasis, cell proliferation, and other normal physiological processes. In the nature, ROS and antioxidants have a stable ratio for cell development. However, it has been shown that in *in vitro* embryo culture conditions, the environment is different than *in vivo* (52) where antioxidants are added to the media to prevent the impact of ROS. Even with the addition of antioxidants, the *in vitro* embryos development still undergoes ROS-mediated oxidative stress (54). Some studies show that culture medium with correct dose of antioxidants protects embryos from oxidative stress (51, 52, 53); however some contrasting evidence has also been presented (55, 56). Overall, it is necessary to control the oxidative stress conditions of embryos to have increased *in vitro* embryo quality.

In the current study, UF-EVs were supplemented to the embryo culture media, which showed significantly improved blastocyst development rates compared to nontreated control. Previous studies have shown that EVs are internalized by embryos (26, 57) being able to reduce ROS levels in embryos (58), which improves blastocyst rates. However, our finding needs further investigation to understand the differences in anti-oxidation–protective effects on embryos, which are supplemented directly to culture medium compared to antioxidants that are carried by EVs. EVs are known to protect their cargo until their transfer to the target cells (59), which could potentially protect the structure of EV cargo proteins from degradation compared to the free proteins in the medium. Therefore, loading appropriate biomolecules needed for embryo development to EVs through the means of EV engineering and supplementing them to culture medium could be an alternative to direct supplementation of biomolecules in the culture media to improve *in vitro* blastocyst rates. Previous studies have investigated the possibility of the use of EVs for loading biomolecules and delivering them to target cells, for example, to improve embryo quality (60), potentially treat or inhibit progression of diseases like Parkinson’s (61, 62), breast cancer (63), or pancreatic ductal adenocarcinoma (64). EVs containing survivin produced by Flk-1+ progenitors have shown to restore cellular homeostasis and prevent diabetes-induced neural defects in the mouse embryo (60). With regards to breast cancer, EVs loaded with let-7a miRNA inhibited tumor development in cultured mouse breast cancer cells (63). Therefore, it is intriguing to use engineered EVs in repeat breeders with the history of early embryo loss to manipulate the uterine microenvironment at the time of embryo transfer which could be another future perspective of use of UF-EVs. Thus, further research is required to develop potential strategies for using EVs as cargo carriers to provide optimal *in vitro* embryo developmental environment.

Despite the efforts made to understand the contribution of EVs to modulate the uterine microenvironment, endometrial functions, and early embryonic development, the optimal EV supplementation conditions and real functional properties of the EVs yet to be fully described since the EV field is still in its infancy. Current study indicated that UF-EVs have functional role on embryo development *in vitro* and highlighted the necessity of adding specific amounts of healthy cow UF-EVs in *in vitro* embryo culture media to achieve greater blastocyst production.

Supplementing UF-EVs from luteal or follicular phase to embryo *in vitro* culture increased the rate of blastocyst production; however the results showed that the blastocyst rate was significantly higher only in the luteal phase UF-EV supplemented group than the control group. The conceptus reaches to the uterus at day 3 to 5 after ovulation (65, 66), which is around the start of luteal phase of the bovine estrous cycle (1). Therefore, the changes in UF-EVs at luteal phase can modulate the uterine environment ideal for embryo development and implantation process. However, in our data, the follicular phase UF-EV–supplemented group had slightly increased blastocyst rates compared to the control group. Around 91% of all UF-EV proteins had no significant differences between the timepoints of the estrous cycle. Many of those proteins have potential roles in promoting uterine microenvironment possibly in paracrine manner and can impact the embryo growth, which might explain the increased blastocyst rates by supplementing UF-EVs in embryo culture. To our knowledge, there are no previous studies researching the impact of cycle-dependent dynamics of UF-EV protein cargo on embryo development. Hence, further studies are warranted to understand UF-EV proteins exact impact on *in vitro* embryo quality and survival specially after freezing and vitrification which may have greater impact on the *in vitro* fertilization and estrous timing of the cows. Furthermore, autologous UF-EV could be an ideal mode to condition the cow endometrium prior to embryo transfer, which may promote the ideal microenvironment enhancing intercellular and intracellular communications.

## Conclusions

Throughout the estrous cycle, the UF-EV proteome undergoes significant changes. Several differentially enriched proteins between day 0, 7, or 16 of the estrous cycle were related to pathways of endometrial remodeling/actin cytoskeleton organization, antioxidant activity, immune and metabolic processes. Comparisons between EVs proteome obtained from natural and hormonal synchronized cycle indicated a shift in protein enrichment. Moreover, bovine UF-EVs have positive impact on *in vitro* blastocyst rate providing an improved microenvironment for embryo growth. The highest rate of blastocysts was produced by adding luteal phase UF-EVs in their optimal concentration. However, future research is required to investigate the differences of UF-EV proteome to identify the optimal EV cargo composition for predicting and improving bovine ART success rates and fertility.

## Data Availability

The data that support the findings of this study is available from the corresponding author upon reasonable request. The mass spectrometry proteomics data have been deposited to the ProteomeXchange Consortium *via* the PRIDE (67) partner repository with the dataset identifier PXD040201.

## Ethics Approval and Consent to Participate

All experiments involving animals were approved by the Committee for Conducting Animal Experiments at the Ministry of Rural Affairs, Estonia (Approval number 200 from 09.07.2021).

## Supplemental data

This article contains supplemental data.

## Conflict of interest

The authors declare no competing interests.
