# Supplementary material for: Uterine Fluid Extracellular Vesicles Proteome Is Altered During the Estrous Cycle
Source: Mol Cell Proteomics. 2023 Sep 9;22(11):100642. doi: 10.1016/j.mcpro.2023.100642 (PMC10641272; doi:10.1016/j.mcpro.2023.100642)
Supplement: Supplementary file 1 [file mmc1.docx]

**Supplementary file 1: Ultrasonographic assessment of ovarian structures in cows included in the study on different timepoints during the oestrous cycle before sampling**.

| **Cow** | **Results of US at day 0** | **Results of US at day 7** | **Results of US at day 16** |
| --- | --- | --- | --- |
| 1 | Right ovary: 25.5 mm follicle, several <5 mm follicles.  Left ovary: several <5 mm follicles. | Right ovary: 20.0 mm follicle, several <5 mm follicles.  Left ovary: 25.5 CL with a hole. | Right ovary: 13.0 mm follicle.  Left ovary: 26.0 mm CL. |
| 2 | Right ovary: 23.2 mm follicle, several <5 mm follicles.  Left ovary: several <5 mm follicles. | Right ovary: 30.0 mm CL with a hole.  Left ovary: two 4-5 mm follicles. | Right ovary: 32.1 mm CL, 24.4 mm follicle.  Left ovary: two <5 mm follicles. |
| 3 | Right ovary: several <5 mm follicles.  Left ovary: 22.1 mm follicle. | Right ovary: 18.9 mm follicle.  Left ovary: 40 mm CL, several <5 mm follicles. | Right ovary: 23.0 mm follicles, several <5 mm follicles.  Left ovary: 36.7 mm CL. |
| 4 | Right ovary: several <5 mm follicles.  Left ovary: 20.7 mm follicle, several <5 mm follicles. | Right ovary: 26.5 mm CL with a hole, 18.0 mm follicle.  Left ovary: several <5 mm follicles. | Right ovary: 26.5 mm CL, 15.0 mm follicle.  Left ovary: 15.0 mm follicle, several <5 mm follicles. |
| 5 | Right ovary: several <5 mm follicles.  Left ovary: several <5 mm follicles. | Right ovary: 29.0 mm CL.  Left ovary: 18.0 mm follicle. | Right ovary: 39.8 mm CL.  Left ovary: 24.5 mm follicle, several <5 mm follicles. |
| 6 | Right ovary: 8.0 mm follicle, several <5 mm follicles.  Left ovary: 10.0 mm follicle, several <5 mm follicles. | Right ovary: several <5 mm follicles.  Left ovary: 33.8 mm CL with a hole, 14.9 mm follicle. | Right ovary: several <5 mm follicles.  Left ovary: 27.8 mm CL with a hole, 15.6 mm follicle, several <5 mm follicles. |

US: ultrasonography; CL: *corpus luteum*.
