# Supplementary material for: Uterine Fluid Extracellular Vesicles Proteome Is Altered During the Estrous Cycle
Source: Mol Cell Proteomics. 2023 Sep 9;22(11):100642. doi: 10.1016/j.mcpro.2023.100642 (PMC10641272; doi:10.1016/j.mcpro.2023.100642)
Supplement: Supplementary file 2 [file mmc2.docx]

**Supplementary file 2: Identified bovine uterine fluid (UF) extracellular vesicles (EV) related protein enrichment or depletion after EV isolation.** One sample of UF protein abundance was measured before and after EV isolation using mass-spectrometry. Thereafter, the comparison was made between the raw UF and isolated UF-EVs showing if EV isolation enriched or depleted proteins.

| **Protein name** | **Protein description** | **Protein abundance before EV isolation*** | **Protein abundance after EV isolation*** | **Status** |
| --- | --- | --- | --- | --- |
| A2M | Alpha-2-macroglobulin | 9.11 | 9.65 | Enriched |
| ACLY | ATP-citrate synthase | 7.32 | 7.55 | Enriched |
| ACTB | Actin beta | 0.00 | 8.26 | Enriched |
| ACTG1 | Actin gamma 1 | 9.34 | 9.19 | Depleted |
| ACTN4 | Actinin alpha 4 | 8.28 | 8.53 | Enriched |
| AHCY | Adenosylhomocysteinase | 8.49 | 8.04 | Depleted |
| ALB | lbumin | 10.38 | 9.58 | Depleted |
| ALDOA | Fructose-bisphosphate aldolase | 8.64 | 7.93 | Depleted |
| ANXA1 | Annexin 1 | 8.70 | 8.11 | Depleted |
| ANXA11 | Annexin 11 | 7.11 | 7.23 | Enriched |
| ANXA2 | Annexin 2 | 8.66 | 8.57 | Depleted |
| ANXA4 | Annexin 4 | 8.12 | 7.93 | Depleted |
| ANXA5 | Annexin 5 | 8.62 | 7.82 | Depleted |
| ANXA6 | Annexin 6 | 6.82 | 6.41 | Depleted |
| ARF1 | ADP-ribosylation factor 1 | 7.67 | 7.44 | Depleted |
| ATP1A1 | Sodium/potassium-transporting ATPase subunit alpha-1 | 7.15 | 7.98 | Enriched |
| CCT2 | T-complex protein 1 subunit beta | 7.68 | 7.77 | Enriched |
| CCT3 | T-complex protein 1 subunit gamma | 7.74 | 7.84 | Enriched |
| CCT5 | T-complex protein 1 subunit epsilon | 7.65 | 7.73 | Enriched |
| CD63 | Cluster determinant 63 | 0.00 | 7.36 | Enriched |
| CD9 | Cluster determinant 9 | 7.53 | 7.92 | Enriched |
| CDC42 | Cell division cycle 42 | 7.78 | 7.69 | Depleted |
| CFL1 | Cofilin-1 | 8.60 | 7.92 | Depleted |
| CLIC1 | Chloride intracellular channel protein 1 | 8.47 | 7.96 | Depleted |
| CLTC | Clathrin heavy chain | 7.85 | 8.55 | Enriched |
| EEF1A1 | Eukaryotic elongation factor 1-alpha 1 | 8.77 | 8.83 | Enriched |
| EEF2 | Eukaryotic elongation factor 2 | 8.33 | 8.24 | Depleted |
| EHD4 | EH domain containing 4 | 6.41 | 0.00 | Depleted |
| ENO1 | Enolase 1 | 8.76 | 7.88 | Depleted |
| EZR | Ezrin | 8.64 | 8.58 | Depleted |
| FASN | Fatty acid synthase | 8.34 | 8.44 | Enriched |
| FLNA | Filamin A | 7.12 | 7.65 | Enriched |
| FN1 | Fibronectin 1 | 7.73 | 8.07 | Enriched |
| GAPDH | Glyceraldehyde-3-phosphate dehydrogenase | 8.66 | 8.45 | Depleted |
| GDI2 | GDP dissociation inhibitor 2 | 8.11 | 7.41 | Depleted |
| GNAI2 | G protein subunit alpha I2 | 7.43 | 7.73 | Enriched |
| GNAS | GNAS complex locus | 0.00 | 7.29 | Enriched |
| GNB1 | G protein subunit beta 1 | 7.36 | 8.19 | Enriched |
| GNB2 | G protein subunit beta 2 | 0.00 | 7.78 | Enriched |
| GSN | Gelsolin | 8.42 | 8.00 | Depleted |
| HSP90AA1 | Heat shock protein 90-alpha family class A member 1 | 8.87 | 9.12 | Enriched |
| HSP90AB1 | Heat shock protein 90-beta family class B member 1 | 8.63 | 8.85 | Enriched |
| HSPA1A | Heat shock protein A member 1A | 7.93 | 7.85 | Depleted |
| HSPA5 | Heat shock protein A member 5 | 8.07 | 8.10 | Enriched |
| HSPA8 | Heat shock protein A member 8 | 8.60 | 8.43 | Depleted |
| ITGA6 | Integrin subunit alpha 6 | 0.00 | 7.17 | Enriched |
| ITGB1 | Integrin subunit beta 1 | 0.00 | 7.56 | Enriched |
| KPNB1 | Karyopherin subunit beta 1 | 7.70 | 7.58 | Depleted |
| LAMP2 | Lysosomal-associated membrane protein 2 | 6.82 | 7.61 | Enriched |
| LDHA | Lactate dehydrogenase A | 8.46 | 7.93 | Depleted |
| LDHB | Lactate dehydrogenase B | 8.44 | 7.93 | Depleted |
| LGALS3BP | Galectin-3-binding protein | 5.74 | 7.41 | Enriched |
| MFGE8 | Lactadherin | 0.00 | 5.84 | Enriched |
| MSN | Moesin | 7.29 | 6.87 | Depleted |
| PDCD6IP | Programmed cell death 6 interacting protein | 7.28 | 7.38 | Enriched |
| PFN1 | Profilin-1 | 8.52 | 7.42 | Depleted |
| PGK1 | Phosphoglycerate kinase 1 | 8.21 | 7.21 | Depleted |
| PKM | Pyruvate kinase | 8.42 | 7.69 | Depleted |
| PPIA | Peptidylprolyl isomerase A | 8.88 | 7.67 | Depleted |
| PRDX1 | Peroxiredoxin-1 | 8.72 | 8.52 | Depleted |
| PRDX2 | Peroxiredoxin-2 | 8.32 | 7.88 | Depleted |
| PTGFRN | Prostaglandin F2 receptor inhibitor | 0.00 | 6.31 | Enriched |
| RAB14 | Small GTP binding protein RAB14 | 7.03 | 7.13 | Enriched |
| RAB1A | Small GTP binding protein RAB1A | 8.02 | 8.10 | Enriched |
| RAB5C | Small GTP binding protein RAB5C | 7.35 | 7.51 | Enriched |
| RAB7A | Small GTP binding protein RAB7A | 7.32 | 7.49 | Enriched |
| RAB8A | Small GTP binding protein RAB8A | 6.96 | 6.71 | Depleted |
| RAC1 | Rac-family small GTPase 1 | 7.67 | 7.49 | Depleted |
| RAN | GTP-binding nuclear protein Ran | 8.16 | 7.77 | Depleted |
| RAP1B | Ras-related protein Rap-1b | 7.34 | 0.00 | Depleted |
| RHOA | Ras homolog family member A | 7.75 | 7.59 | Depleted |
| SDCBP | Syndecan binding protein | 7.22 | 7.72 | Enriched |
| SLC16A1 | Solute carrier family 16-member 1 | 0.00 | 6.75 | Enriched |
| STOM | Stomatin | 0.00 | 7.14 | Enriched |
| TCP1 | T-complex protein 1 | 7.57 | 7.73 | Enriched |
| THBS1 | Thrombospondin-1 | 6.83 | 7.77 | Enriched |
| TKT | Transketolase | 8.53 | 7.77 | Depleted |
| TPI1 | Triosephosphate isomerase 1 | 8.47 | 7.39 | Depleted |
| TSG101 | Tumor susceptibility gene 101 | 0.00 | 6.80 | Enriched |
| TUBA1A | Tubulin alpha chain | 8.88 | 8.96 | Enriched |
| TUBA1B | Tubulin alpha-1B chain | 8.76 | 8.74 | Depleted |
| TUBA1C | Tubulin alpha-1C chain | 7.19 | 7.06 | Depleted |
| UBA1 | Ubiquitin-like modifier-activating enzyme 1 | 8.10 | 7.50 | Depleted |
| VCP | Valosin containing protein | 8.16 | 8.37 | Enriched |
| YWHAB | 14-3-3 protein beta/alpha | 8.15 | 7.68 | Depleted |
| YWHAE | 14-3-3 protein epsilon | 8.32 | 7.86 | Depleted |
| YWHAG | 14-3-3 protein gamma | 8.01 | 7.74 | Depleted |
| YWHAH | 14-3-3 protein eta | 7.67 | 7.22 | Depleted |
| YWHAQ | 14-3-3 protein theta | 8.06 | 7.83 | Depleted |
| YWHAZ | 14-3-3 protein zeta/delta | 8.60 | 8.10 | Depleted |

* Log10 transformed values
