# Supplementary material for: Uterine Fluid Extracellular Vesicles Proteome Is Altered During the Estrous Cycle
Source: Mol Cell Proteomics. 2023 Sep 9;22(11):100642. doi: 10.1016/j.mcpro.2023.100642 (PMC10641272; doi:10.1016/j.mcpro.2023.100642)
Supplement: Supplementary file 3 [file mmc3.docx]

**Supplementary file 3: Differentially enriched uterine fluid extracellular vesicle proteins between day 0 (D0) and day 7 (D7), D7 and day 16 (D16), and D0 and D16.**

| **Gene Name** | **Protein name** | **Log2 fold change** | | | ***P*-adjusted values** | | | **Significant** | | | **Putative function*** |
| --- | --- | --- | --- | --- | --- | --- | --- | --- | --- | --- | --- |
|  |  | **D0 vs D16** | **D0 vs D7** | **D7 vs D16** | **D0 vs D16** | **D0 vs D7** | **D7 vs D16** | **D0 vs D16** | **D0 vs D7** | **D7 vs D16** |  |
| A0A3Q1MDT7 | Histone H4 | -3.60 | 1.45 | -5.05 | 0.04 | 0.70 | 0.04 | T | F | T | - |
| ABHD14B | Abhydrolase Domain Containing 14B | -1.65 | -1.01 | -0.64 | 0.03 | 0.48 | 0.36 | T | F | F | positive regulation of transcription from RNA polymerase II promoter |
| ACE | Angiotensin-converting enzyme | -1.57 | -1.60 | 0.03 | 0.05 | 0.23 | 0.98 | T | F | F | beta-amyloid metabolic process, negative regulation of gap junction assembly, neutrophil mediated immunity, cellular metabolic process, mononuclear cell proliferation, hormone catabolic process, regulation of vasoconstriction |
| ACTC1 | Actin alpha cardiac muscle 1 | -1.65 | -0.14 | -1.51 | 0.04 | 0.97 | 0.12 | T | F | F | apoptotic process, actin filament-based movement, response to ethanol, positive regulation of gene expression, negative regulation of apoptotic process, actomyosin structure organization |
| AGRN | Agrin | 2.86 | 0.27 | 2.59 | 0.04 | 0.97 | 0.13 | T | F | F | signal transduction, glycosaminoglycan metabolic process, extracellular matrix reorganization, positive regulation of GTPase activity, carbohydrate metabolic process |
| AKR1A1 | Aldo-keto reductase family 1 member A1 | -2.34 | -0.56 | -1.78 | 0.02 | 0.85 | 0.11 | T | F | F | D-glucuronate catabolic process, aldehyde catabolic process, L-ascorbic acid biosynthesis process, oxidation-reduction process, glucose metabolic process |
| AKR1B1 | Aldo-keto reductase family 1 member B1 | -1.99 | 0.68 | -2.67 | 0.03 | 0.73 | 0.02 | T | F | T | response to stress, oxidation-reduction process, carbohydrate metabolic process, fructose biosynthesis process, steroid metabolic process, C21-steroid hormone biosynthetic process |
| ALPL | Alkaline phosphatase tissue-nonspecific isozyme | -3.34 | -0.88 | -2.46 | 0.02 | 0.75 | 0.10 | T | F | F | cellular response to organic cyclic compound, dephosphorylation, response to glucocorticoid, response to lipopolysaccharide |
| ANXA1 | Annexin A1 | -1.68 | -1.54 | -0.15 | 0.03 | 0.20 | 0.88 | T | F | F | calcium ion transmembrane transport, negative regulation of catalytic activity, signal transduction, insulin secretion, cellular response to hydrogen peroxide, response to interleukin-1, positive regulation of prostaglandin biosynthetic process, regulation of cell proliferation, positive regulation of neutrophil apoptotic process, movement of cell or subcellular component, prolactin secretion, inflammatory response, negative regulation of apoptotic process, response to oestradiol, cell surface receptor signalling pathway |
| AP2A1 | AP-2 complex subunit alpha-1 | 3.82 | 1.21 | 2.61 | 0.04 | 0.81 | 0.22 | T | F | F | Intracellular protein transport, epidermal growth factor receptor signalling pathway. |
| AP2B1 | AP-2 complex subunit beta-1 | 1.60 | 0.52 | 1.09 | 0.03 | 0.76 | 0.19 | T | F | F | Intracellular protein transport, vesicle-mediated transport, endocytosis, epidermal growth factor receptor signalling pathway |
| AP2M1 | AP-2 complex subunit mu-1 | 1.59 | 0.42 | 1.17 | 0.04 | 0.88 | 0.18 | T | F | F | Intracellular protein transport, endocytosis, negative regulation of protein localization to plasma membrane. Gap junction degradation. |
| ARHGAP27 | Rho GTPase activating protein 27 | -2.27 | -1.21 | -1.06 | 0.03 | 0.52 | 0.32 | T | F | F | - |
| ARHGDIA | Rho GDP-dissociation inhibitor alpha | -2.04 | -0.85 | -1.19 | 0.02 | 0.52 | 0.17 | T | F | F | positive regulation of GTPase activity, regulation of protein localization, Rho protein signal transduction, semaphorin-plexin signalling pathway, negative regulation of cell adhesion, negative regulation of cell migration |
| ARHGDIB | Rho GDP-dissociation inhibitor beta | -2.31 | 0.14 | -2.46 | 0.03 | 0.97 | 0.05 | T | F | T | multicellular organismal development, Rho protein signal transduction, small GTPase mediated signal transduction, immune response, movement of cell or subcellular component, regulation of catalytic activity, actin cytoskeleton organization, negative regulation of cell adhesion, negative regulation of trophoblast cell migration |
| ARRDC1 | Arrestin domain containing 1 | -2.75 | -0.94 | -1.81 | 0.02 | 0.63 | 0.13 | T | F | F | - |
| AZU1 | Azurocidin 1 | -4.92 | 0.11 | -5.04 | 0.01 | 0.97 | 0.02 | T | F | T | protein processing, immune response, monocyte activation, macrophage chemotaxis, negative regulation of apoptotic process, protein kinase C-activating G-protein coupled receptor signalling pathway, positive regulation of cell adhesion |
| B2M | Beta-2-microglobulin | -1.88 | -1.18 | -0.70 | 0.03 | 0.48 | 0.39 | T | F | F | iron ion homeostasis, protein refolding, positive regulation of T cell mediated cytotoxicity, antigen processing and presentation of exogenous protein antigen via MHC class Ib, TAP-dependent |
| BAIAP2 | Brain-specific angiogenesis inhibitor 1-associated protein 2 | -2.26 | -0.94 | -1.32 | 0.03 | 0.63 | 0.25 | T | F | F | insulin receptor signalling pathway, plasma membrane organization, regulation of actin cytoskeleton organization, regulation of cell shape, small GTPase mediated signal transduction, innate immune response |
| BAIAP2L1 | BAI1 associated protein 2 like 1 | -2.12 | -1.72 | -0.40 | 0.04 | 0.33 | 0.74 | T | F | F | actin crosslink formation, plasma membrane organization, insulin receptor signalling pathway, positive regulation of actin cytoskeleton reorganization, regulation of insulin receptor signalling pathway |
| BCAM | Basal cell adhesion molecule | -0.51 | 1.67 | -2.17 | 0.46 | 0.23 | 0.05 | F | F | T | cell adhesion, signal transduction, cell-matrix adhesion |
| BLVRB | Biliverdin reductase B | -1.84 | -0.33 | -1.50 | 0.02 | 0.93 | 0.07 | T | F | F | oxidation-reduction process, heme catabolic process, porphyrin-containing compound metabolic process |
| CA2 | Carbonic anhydrase 2 | -3.57 | 0.07 | -3.64 | 0.05 | 0.98 | 0.11 | T | F | F | bicarbonate transport, response to zinc ion, regulation of intracellular pH, response to oestrogen, regulation of anion transport, angiotensin-activated signalling pathway, one-carbon metabolic process, small molecule metabolic process |
| CAB39 | Calcium-binding protein 39 | -1.84 | -1.69 | -0.15 | 0.04 | 0.26 | 0.92 | T | F | F | positive regulation of peptidyl-threonine phosphorylation, activation of protein kinace activity, intracellular signal transduction, insulin receptor signalling pathway, cell cycle arrest |
| CALM | Calmodulin | -1.69 | -0.84 | -0.84 | 0.04 | 0.57 | 0.31 | T | F | F | membrane organization, innate immune response, glucose and carbohydrate metabolic process, small GTPase mediated signal transduction, response to calcium ion |
| CAPG | Macrophage-capping protein | -2.84 | -0.98 | -1.86 | 0.02 | 0.63 | 0.13 | T | F | F | protein complex assembly, extracellular matrix disassembly |
| CAPRIN1 | Cell cycle associated protein 1 | 1.57 | 0.72 | 0.85 | 0.05 | 0.67 | 0.32 | T | F | F | negative regulation of translation |
| CATHL1 | Cathelicidin-1 | -6.49 | -1.87 | -4.62 | 0.00 | 0.50 | 0.01 | T | F | T | defence response to bacterium |
| CATHL7 | Cathelicidin-7 | -4.42 | -1.83 | -2.59 | 0.01 | 0.52 | 0.13 | T | F | F | - |
| CDC42 | Cell division cycle protein 42 | -1.58 | -0.80 | -0.78 | 0.04 | 0.59 | 0.33 | T | F | F | cell differentiation, small GTPase mediated signal transduction, positive regulation of cytokinesis, positive regulation of substrate adhesion-dependent cell spreading |
| CFDP2 | Craniofacial development protein 2 | 2.69 | 1.07 | 1.62 | 0.05 | 0.72 | 0.27 | T | F | F | - |
| CHMP2A | Charged multivesicular body protein 2A | -2.09 | -0.41 | -1.68 | 0.04 | 0.96 | 0.16 | T | F | F | protein transport, membrane invagination, protein polymerization, cell separation after cytokinesis, regulation of mitotic spindle assembly, positive regulation of exosomal secretion, membrane organization, negative regulation of cell death, endosomal transport |
| CLTC | Clathrin heavy chain 1 | 1.74 | 0.88 | 0.86 | 0.03 | 0.53 | 0.30 | T | F | F | receptor internalization, intracellular protein transport, mitotic nuclear division, membrane organization, transferrin transport. Gap junction degradation, lysosome vesicle biogenesis. |
| COL6A3 | Collagen type VI alpha 3 chain | -3.84 | -0.71 | -3.13 | 0.04 | 0.96 | 0.15 | T | F | F | cell adhesion, extracellular matrix organization |
| COPA | Coatomer subunit alpha | 2.27 | 0.66 | 1.61 | 0.05 | 0.87 | 0.22 | T | F | F | intracellular protein transport, membrane organization |
| CZIB | CXXC motif-containing zinc-binding protein | 2.06 | -0.11 | 2.16 | 0.04 | 0.97 | 0.08 | T | F | F | - |
| DBI | Acyl-CoA-binding protein | -2.50 | 0.09 | -2.59 | 0.03 | 0.97 | 0.05 | T | F | T | triglyceride metabolic process, phosphatidylcholine acyl-chain remodelling, transport |
| DDX17 | DEAD-box helicase 17 | 2.02 | 0.84 | 1.17 | 0.05 | 0.70 | 0.29 | T | F | F | RNA processing, positive regulation of intracellular oestrogen receptor signalling pathway |
| DDX31 | DEAD-box helicase 31 | 2.39 | 0.40 | 1.99 | 0.04 | 0.97 | 0.13 | T | F | F | - |
| DEFB13 | Beta-defensin 13 | -2.99 | -0.11 | -2.89 | 0.03 | 0.97 | 0.08 | T | F | F | - |
| DEFB2 | Beta-defensin 2 | -3.89 | 0.11 | -4.00 | 0.03 | 0.97 | 0.05 | T | F | T | - |
| DHX15 | DEAH-box helicase 15 | 1.74 | 0.70 | 1.04 | 0.03 | 0.67 | 0.24 | T | F | F | RNA splicing, mRNA processing |
| DHX9 | DEAX-box helicase 9 | 2.45 | 0.83 | 1.62 | 0.03 | 0.69 | 0.17 | T | F | F | positive regulation of type I interferon production, gene expression, RNA processing, innate immune response, cellular response to heat |
| EDC4 | Enhancer of mRNA decapping 4 | 1.62 | 1.92 | -0.30 | 0.01 | 0.02 | 0.61 | T | T | F | gene expression |
| EHD1 | EH-domain containing protein 1 | -2.78 | -1.22 | -1.56 | 0.02 | 0.52 | 0.19 | T | F | F | intracellular protein transport, cholesterol homeostasis, endocytosis, low-density lipoprotein particle clearance |
| EHD4 | EH-domain containing protein 4 | -2.42 | -1.28 | -1.14 | 0.05 | 0.59 | 0.37 | T | F | F | positive regulation of peptidyl-tyrosine phosphorylation, cellular response to growth factor stimulus, regulation of endocytosis |
| EIF2S2 | Eukaryotic translation initiation factor 2 subunit 2 | 2.53 | 0.84 | 1.69 | 0.04 | 0.78 | 0.22 | T | F | F | in utero embryonic development, translation, cellular metabolic process, gene expression |
| EIF2S3 | Eukaryotic translation initiation factor 2 subunit 3 | 2.75 | 0.99 | 1.76 | 0.05 | 0.79 | 0.26 | T | F | F | transmembrane transport, gene expression, cellular protein metabolic process, translation. |
| ELANE | Elastase | -5.33 | 0.13 | -5.46 | 0.02 | 0.98 | 0.05 | T | F | T | proteolysis, defence mechanism to bacterium, negative regulation of interleukin-8 biosynthetic process, negative regulation of chemotaxis, positive regulation of MAP kinase activity, negative regulation of inflammatory response, phagocytosis, response to lipopolysaccharide, cellular calcium ion homeostasis, extracellular matrix organization, protein catabolic process, leukocyte migration |
| EMB | Embigin | -1.41 | 0.65 | -2.06 | 0.07 | 0.72 | 0.05 | F | F | T | cell adhesion, plasma membrane lactate transport |
| EML4 | Echinoderm microtubule associated protein like 4 | 3.65 | 1.40 | 2.25 | 0.01 | 0.54 | 0.13 | T | F | F | mitotic nuclear division, microtubule-based process. |
| EPRS1 | Glutamyl-prolyl-tRNA synthetase | 1.97 | 0.79 | 1.18 | 0.03 | 0.62 | 0.21 | T | F | F | gene expression, protein complex assembly, cellular response to interferon-gamma, negative regulation of translation. |
| EPS8 | Epidermal growth factor receptor pathway substrate 8 | -3.99 | -2.12 | -1.88 | 0.01 | 0.32 | 0.19 | T | F | F | cell proliferation, actin cytoskeleton reorganization, Ras protein signal transduction, cell proliferation, signal transduction, regulation of actin filament length, actin polymerization-dependent cell motility, exit from mitosis |
| EPS8L1 | EPS8-like 1 | -2.73 | -1.50 | -1.23 | 0.03 | 0.51 | 0.31 | T | F | F | positive regulation of GTPase activity, regulation of Rho protein signal transduction, positive regulation of ruffle assembly |
| EPS8L2 | EPS8-like 2 | -3.03 | -0.95 | -2.08 | 0.03 | 0.74 | 0.16 | T | F | F | positive regulation of GTPase activity, Rho protein signal transduction |
| FAM120A | Family with sequence similarity 120A | 1.89 | 0.63 | 1.26 | 0.02 | 0.64 | 0.13 | T | F | F | RNA binding |
| FAU | 40S ribosomal protein S30 | 1.91 | 0.57 | 1.34 | 0.04 | 0.86 | 0.21 | T | F | F | - |
| FCGBP | Fc fragment of IgG binding protein | -3.23 | -3.64 | 0.41 | 0.02 | 0.04 | 0.77 | T | T | F | protein binding |
| FLNB | Filamin B | 1.51 | 0.83 | 0.69 | 0.04 | 0.55 | 0.37 | T | F | F | cell differentiation, signal transduction, cytokine-mediated signalling pathway, actin cytoskeleton organization. |
| FXR1 | Fragile X mental retardation syndrome-related protein 1 | 2.72 | 0.85 | 1.87 | 0.02 | 0.69 | 0.13 | T | F | F | cell differentiation, apoptotic process, negative regulation of translation. |
| G5E5A7 | Uncharacterized protein | 3.08 | 1.02 | 2.06 | 0.00 | 0.51 | 0.05 | T | F | T | - |
| GIPC2 | PDZ domain-containing protein GIPC family member 2 | -3.27 | -0.62 | -2.65 | 0.01 | 0.87 | 0.05 | T | F | F | - |
| GNG12 | Guanine nucleotide-binding protein subunit gamma-12 | -2.08 | -1.18 | -0.90 | 0.01 | 0.26 | 0.22 | T | F | F | G-protein coupled receptor signalling pathway, response to lipopolysaccharide, cellular response to glucagon stimulus, signal transduction |
| GNG5 | Guanine nucleotide-binding protein subunit gamma-5 | -1.74 | -0.84 | -0.91 | 0.04 | 0.60 | 0.31 | T | F | F | G-protein coupled receptor signalling pathway, cellular response to glucagon stimulus, small molecule metabolic process |
| GRP | Gastrin-releasing peptide | -2.01 | -0.74 | -1.27 | 0.04 | 0.74 | 0.26 | T | F | F | - |
| GSTP1 | Glutathione S-transferase P 1 | -1.65 | -0.99 | -0.66 | 0.02 | 0.48 | 0.33 | T | F | F | negative regulation of interleukin-1 beta production, negative regulation of nitric-oxide synthase biosynthesis process, glutathione metabolic process |
| H1-0 | Histone H1.0 | 1.67 | 0.61 | 1.06 | 0.04 | 0.75 | 0.25 | T | F | F | - |
| H2BC12 | H2B clustered histone 12 | -2.93 | 0.93 | -3.86 | 0.04 | 0.79 | 0.03 | T | F | T | - |
| HIST2H2AC | Histone H2A type 2-C | -2.83 | 0.49 | -3.32 | 0.04 | 0.97 | 0.06 | T | F | F | - |
| HIST2H3D | Histone H3.2 | -3.77 | 2.20 | -5.97 | 0.05 | 0.55 | 0.03 | F | F | T | DNA methylation on cytosine, nucleosome assembly, gene expression, small GTPase mediated signal transduction |
| HP | Haptoglobin | -6.43 | -1.79 | -4.64 | 0.00 | 0.57 | 0.04 | T | F | T | immune system process, defence response to bacterium, acute-phase response, antioxidant activity |
| HSPG2 | Heparan sulfate proteoglycan 2 | 3.90 | 1.72 | 2.18 | 0.03 | 0.59 | 0.25 | T | F | F | protein localization, lipoprotein metabolic process, extracellular matrix reorganization, glycosaminoglycan metabolic process. Non-integrin membrane-ECM interactions, integrin cell surface interactions, lamin interactions. |
| HTRA1 | HTRA serine peptidase 1 | -7.60 | -2.77 | -4.82 | 0.00 | 0.26 | 0.02 | T | F | T | proteolysis, regulation of cell growth, positive regulation of epithelial cell proliferation, negative regulation of transforming growth factor beta receptor signalling pathway |
| HYOU1 | Hypoxia up-regulated 1 | -1.95 | 0.43 | -2.38 | 0.04 | 0.94 | 0.05 | T | F | T | IRE1-mediated unfolded protein response, cellular protein metabolic process, endoplasmic reticulum unfolded protein response, receptor-mediated endocytosis, cellular response to hypoxia |
| IARS1 | Isoleucyl-tRNA synthetase 1 | 4.04 | 1.66 | 2.38 | 0.02 | 0.59 | 0.19 | T | F | F | - |
| ILF2 | Interleukin enhancer binding factor 2 | 2.23 | 0.76 | 1.47 | 0.05 | 0.81 | 0.25 | T | F | F | positive regulation of transcription, DNA-templated, immune response |
| IST1 | IST1 homolog | -1.84 | -0.49 | -1.35 | 0.04 | 0.87 | 0.18 | T | F | F | cell division, establishment of protein localization, cytokinesis, positive regulation of proteolysis |
| KARS1 | Lysine--tRNA ligase | 2.53 | 1.10 | 1.43 | 0.03 | 0.59 | 0.23 | T | F | F | tRNA processing, gene expression, cytosolic tRNA aminoacylation. |
| KIF1C | Kinesin family member 1C | -1.68 | -1.13 | -0.55 | 0.04 | 0.50 | 0.53 | T | F | F | microtubule-based movement, cytoskeleton-dependent intracellular transport, retrograde vesicle-mediated transport |
| KIF2A | Kinesin family member 2A | 2.20 | 2.22 | -0.02 | 0.03 | 0.13 | 0.99 | T | F | F | cell division, cell differentiation, metabolic process, small GTPase mediated signal transduction, mitotic spindle assembly |
| LCN2 | Lipocalin 2 | -4.05 | -1.21 | -2.84 | 0.04 | 0.86 | 0.22 | T | F | F | cellular iron ion homeostasis, innate immune response, apoptotic process, transmembrane transport, ion transport |
| LGALS3BP | Galectin-3-binding protein | -4.55 | -3.16 | -1.39 | 0.02 | 0.26 | 0.42 | T | F | F | cell adhesion, receptor-mediated endocytosis |
| LOC112441458 | Uncharacterized protein | -2.62 | 0.17 | -2.78 | 0.03 | 0.97 | 0.05 | T | F | T | - |
| LOC784254 | Alpha-carbonic anhydrase domain-containing protein | -2.71 | -0.11 | -2.61 | 0.03 | 0.97 | 0.07 | T | F | F | - |
| LY6G6C | Lymphocyte antigen 6 complex locus protein G6C | -4.00 | -0.70 | -3.31 | 0.01 | 0.89 | 0.05 | T | F | T | - |
| MAGED2 | MAGE family member D2 | -1.50 | -1.45 | -0.06 | 0.04 | 0.21 | 0.96 | T | F | F | - |
| MAP4 | Microtubule-associated protein 4 | 4.75 | 1.28 | 3.47 | 0.03 | 0.86 | 0.16 | T | F | F | cell division, microtubule sliding, mitotic spindle organization |
| MAP7 | Microtubule associated protein 7 | 2.47 | 1.61 | 0.86 | 0.03 | 0.48 | 0.44 | T | F | F | establishment or maintenance of cell polarity, protein localization to plasma membrane, response to osmotic stress, microtubule cytoskeleton organization |
| MET | Hepatocyte growth factor receptor | -4.56 | -1.15 | -3.41 | 0.02 | 0.79 | 0.10 | T | F | F | signal transduction, placenta development, positive regulation of transcription from RNA polymerase II promoter, cell proliferation, cell surface receptor signalling pathway, positive regulation of epithelial cell chemotaxis, activation of MAPK activity, endothelial cell morphogenesis. Sema4D mediated inhibition of cell attachment and migration. |
| MPO | Myeloperoxidase | -2.64 | 1.05 | -3.69 | 0.05 | 0.74 | 0.05 | T | F | T | defence response, low-density lipoprotein particle remodelling, oxidation-reduction process, negative regulation of apoptotic process, response to lipopolysaccharide, response to oxidative stress, response to mechanical stimulus |
| MUC13 | Mucin 13 | -2.32 | 1.06 | -3.37 | 0.04 | 0.63 | 0.02 | T | F | T | protein O-linked glycosylation, post-translational protein modification, cellular protein metabolic process |
| MUC4 | Mucin 4 | -2.80 | -3.13 | 0.33 | 0.03 | 0.10 | 0.82 | T | F | F | cell-matrix adhesion, protein O-linked glycosylation, post-translational protein modification, cellular protein metabolic process |
| MYH10 | Myosin-10 | -0.05 | -2.27 | 2.22 | 0.96 | 0.11 | 0.05 | F | F | T | cell proliferation, plasma membrane repair, in utero embryonic development, regulation of cell shape, cell adhesin, exocytosis, mitotic cytokinesis |
| NCL | Nucleolin | 2.49 | 0.85 | 1.64 | 0.02 | 0.64 | 0.13 | T | F | F | positive regulation of transcription of nuclear large rRNA transcript from RNA polymerase I promoter, angiogenesis |
| NDRG1 | N-myc downstream regulated 1 | 1.87 | -0.11 | 1.98 | 0.00 | 0.97 | 0.00 | T | F | T | cellular response to hypoxia, DNA damage response, mast cell activation, regulation of cell proliferation |
| NPC1 | NPC intracellular cholesterol transporter 1 | -1.72 | -1.68 | -0.05 | 0.03 | 0.13 | 0.96 | T | F | F | protein glycosylation, cholesterol metabolic process, endocytosis, establishment of protein localization to membrane, cellular response to steroid hormone stimulus, signal transduction |
| OLFM4 | Olfactomedin 4 | -3.88 | -0.11 | -3.77 | 0.05 | 0.98 | 0.13 | T | F | F | cell adhesion, negative regulation of immune response, negative regulation of apoptotic process |
| OXTR | Oxytocin receptor | 2.23 | 2.13 | 0.09 | 0.04 | 0.23 | 0.96 | T | F | F | maternal behaviour, female pregnancy, response to oestradiol, response to cytokine, cell surface receptor signalling pathway, cellular response to hormone stimulus, response to progesterone |
| PAK1IP1 | P21-activated protein kinase-interacting protein 1 | -1.52 | -1.46 | -0.06 | 0.03 | 0.20 | 0.96 | T | F | F | - |
| PDCD10 | Programmed cell death 10 | -2.83 | -2.16 | -0.66 | 0.01 | 0.11 | 0.44 | T | F | F | negative regulation of gene expression, positive regulation of cell migration, regulation of Rho protein signal transduction, positive regulation of protein serine/threonine kinase activity, positive regulation of cell proliferation, response to hydrogen peroxide, positive regulation of MAP kinase activity, positive regulation of peptidyl-serine phosphorylation, negative regulation of apoptotic process, protein stabilization |
| PEBP1 | Phosphatidylethanolamine-binding protein 1 | -2.18 | -1.49 | -0.69 | 0.03 | 0.35 | 0.45 | T | F | F | negative regulation of endopeptidase activity |
| PGAM1 | Phosphoglycerate mutase 1 | -1.71 | -0.64 | -1.07 | 0.04 | 0.70 | 0.23 | T | F | F | glycolytic process, dephosphorylation, carbohydrate metabolic process |
| PGK1 | Phosphoglycerate kinase 1 | -1.57 | -0.53 | -1.04 | 0.04 | 0.76 | 0.22 | T | F | F | glycolytic process, epithelial cell differentiation, phosphorylation |
| PGLYRP1 | Peptidoglycan recognition protein 1 | -3.53 | 0.15 | -3.68 | 0.04 | 0.97 | 0.07 | T | F | F | detection of bacterium, negative regulation of interferon-gamma production, pattern recognition receptor signalling pathway, negative regulation of inflammatory response, innate immune response, peptidoglycan catabolic process |
| PGR | Progesterone receptor | 0.23 | -2.57 | 2.80 | 0.75 | 0.04 | 0.02 | F | T | T | - |
| PKM | Pyruvate kinase | -1.54 | -0.57 | -0.97 | 0.04 | 0.71 | 0.24 | T | F | F | small molecule metabolic process, glucose metabolic process, programmed cell death, phosphorylation, carbohydrate metabolic process |
| PPIA | Peptidyl-prolyl cis-trans isomerase A | -1.67 | -0.82 | -0.85 | 0.03 | 0.55 | 0.29 | T | F | F | protein folding, protein peptidyl-prolyl isomerization, leukocyte migration, lipid particle organization, positive regulation of protein secretion |
| PRDX6 | Peroxiredoxin-6 | -2.60 | -0.98 | -1.63 | 0.01 | 0.52 | 0.09 | T | F | F | oxidation-reduction process, response to reactive oxygen species, phospholipid catabolic process, hydrogen peroxide catabolic process |
| PRSS8 | Serine protease 8 | -2.80 | -1.22 | -1.58 | 0.02 | 0.55 | 0.21 | T | F | F | proteolysis, positive regulation of sodium ion transport |
| PTPRJ | Protein tyrosine phosphatase receptor type J | -2.65 | -0.22 | -2.44 | 0.02 | 0.97 | 0.06 | T | F | F | Negative regulation of T cell receptor signalling pathway, regulation of cell adhesion, vasculogenesis, negative regulation of cell migration and cell proliferation. |
| Q6B411 | Lysozyme C | -5.15 | 0.40 | -5.55 | 0.00 | 0.97 | 0.00 | T | F | T | - |
| QARS1 | Glutamine-tRNA synthetase 1 | 2.32 | 0.96 | 1.36 | 0.04 | 0.69 | 0.27 | T | F | F | gene expression, tRNA aminoacylation for protein translation. |
| RAB8A | Ras-related protein Rab-8A | -1.83 | -1.07 | -0.76 | 0.04 | 0.52 | 0.41 | T | F | F | intracellular protein transport, synaptic vesicle exocytosis, cellular response to insulin stimulus, protein localization to plasma membrane, rab protein signal transduction |
| RAC1 | Rac family small GTPase 1 | -1.82 | -0.79 | -1.03 | 0.04 | 0.68 | 0.29 | T | F | F | cell motility, cell-cell junction organization, G-protein coupled receptor signalling pathway, positive regulation of substrate adhesion-dependent cell spreading, small GTPase mediated signal transduction, positive regulation of neutrophil chemotaxis, positive regulation of protein phosphorylation, positive regulation of focal adhesion assembly, negative regulation of interleukin-23 production, regulation of cell migration |
| RALA | RAS like proto-oncogene A | -2.11 | -0.51 | -1.60 | 0.03 | 0.87 | 0.13 | T | F | F | exocytosis, actin cytoskeleton reorganization, chemotaxis, cytokinesis, membrane organization, neurotrophin TRK receptor signalling pathway, Ras protein signal transduction |
| RALB | RAS like proto-oncogene B | -2.07 | -0.86 | -1.21 | 0.04 | 0.67 | 0.26 | T | F | F | positive regulation of protein serine/threonine kinase activity, apoptotic process, regulation of exocyst assembly, regulation of protein binding, neutrophin TRK receptor signalling pathway, cellular response to exogenous dsRNA, Ras protein signal transduction, cytokinesis |
| RAP1B | Ras-related protein Rap-1b | -2.19 | -1.10 | -1.09 | 0.01 | 0.42 | 0.18 | T | F | F | negative regulation of synaptic vesicle exocytosis, Ras protein signal transduction, cell proliferation, regulation of cell junction assembly, regulation of establishment of cell polarity, cellular response to cAMP |
| RARS1 | Arginyl - tRNA synthetase 1 | 1.74 | 0.79 | 0.95 | 0.04 | 0.60 | 0.27 | T | F | F | gene expression, tRNA aminoacylation for protein translation. |
| RBP4 | Retinol-binding protein 4 | -2.04 | -0.81 | -1.23 | 0.03 | 0.67 | 0.24 | T | F | F | response to insulin, gluconeogenesis, glucose homeostasis |
| RETN | Resistin | -3.52 | -0.88 | -2.64 | 0.00 | 0.67 | 0.05 | T | F | T | response to insulin, positive regulation of collagen metabolic process, positive regulation of progesterone secretion, response to mechanical stimulus, positive regulation of synaptic transmission |
| RHOA | Ras homolog family member A | -1.56 | -0.80 | -0.76 | 0.04 | 0.59 | 0.33 | T | F | F | negative chemotaxis, mitotic spindle assembly, small GTPase mediated signal transduction, regulation of cell migration, positive regulation of cytokinesis. PI3K/AKT activation, VEGFA-VEGFR2 pathway, RHO GTPase cycle |
| RHOB | Ras homolog family member B | 1.59 | -0.11 | 1.70 | 0.01 | 0.97 | 0.01 | T | F | T | small GTPase mediated signal transduction, cell differentiation, protein transport, negative regulation of cell cycle, apoptotic process, cell adhesion |
| RP2 | Retinitis pigmentosa 2 | -1.59 | -1.20 | -0.39 | 0.04 | 0.42 | 0.64 | T | F | F | protein transport, cytoskeleton organization, cell morphogenesis, GTP biosynthetic process, post-chaperonin tubulin folding pathway |
| RPL10 | 60S ribosomal protein L10 | 1.86 | 0.35 | 1.50 | 0.04 | 0.96 | 0.16 | T | F | F | translation, cellular protein metabolic process, gene expression |
| RPL23 | 60S ribosomal protein L23 | 3.42 | 0.52 | 2.90 | 0.03 | 0.97 | 0.11 | T | F | F | translation, cellular protein metabolic process, gene expression |
| RPL27A | 60S ribosomal protein L27a | 2.04 | 0.29 | 1.74 | 0.04 | 0.97 | 0.14 | T | F | F | translation, cellular protein metabolic process, gene expression |
| RPL38 | 60S ribosomal protein L38 | 2.68 | 0.62 | 2.06 | 0.03 | 0.91 | 0.14 | T | F | F | translation, cellular protein metabolic process, gene expression |
| RPL4 | 60S ribosomal protein L4 | 2.28 | 0.36 | 1.92 | 0.03 | 0.97 | 0.11 | T | F | F | translation, cellular protein metabolic process, gene expression |
| RPLP1 | 60S acidic ribosomal protein P1 | 2.49 | 0.50 | 1.99 | 0.05 | 0.96 | 0.17 | T | F | F | translation, cellular protein metabolic process, gene expression |
| S100A11 | S100 calcium binding protein A11 | -1.51 | -1.32 | -0.19 | 0.04 | 0.26 | 0.82 | T | F | F | negative regulation of DNA replication, signal transduction, negative regulation of cell proliferation |
| S100A12 | S100 calcium binding protein A12 | -3.41 | -0.37 | -3.04 | 0.03 | 0.97 | 0.08 | T | F | F | mast cell degranulation, innate immune response, inflammatory response |
| S100A8 | S100 calcium binding protein A8 | -4.54 | -0.66 | -3.88 | 0.02 | 0.97 | 0.08 | T | F | F | innate immune response, leukocyte migration in inflammatory response, neutrophil aggregation, neutrophil chemotaxis, activation of cystine-type endopeptidase activity involved in apoptotic process |
| S100A9 | S100 calcium binding protein A9 | -4.75 | -0.57 | -4.18 | 0.02 | 0.97 | 0.07 | T | F | F | innate immune response, leukocyte migration in inflammatory response, neutrophil aggregation, neutrophil chemotaxis, cell-cell signalling |
| SDF4 | Stromal cell derived factor 4 | -6.06 | -2.27 | -3.78 | 0.02 | 0.57 | 0.13 | T | F | F | calcium ion-dependent exocytosis, UV protection, response to ethanol, protein binding, calcium ion binding |
| SEMA7A | Semaphorin 7A | -4.47 | -1.23 | -3.25 | 0.03 | 0.86 | 0.17 | T | F | F | - |
| SERBP1 | SERPINE1 mRNA binding protein 1 | 2.56 | 0.77 | 1.79 | 0.02 | 0.74 | 0.13 | T | F | F | regulation of mRNA stability, regulation of apoptotic process |
| SERPINB1 | Serpin family B member 9 | -3.92 | -1.21 | -2.71 | 0.00 | 0.52 | 0.05 | T | F | T | negative regulation of endopeptidase activity |
| SH2D4A | SH2 domain containing 4A | 2.24 | 0.56 | 1.69 | 0.03 | 0.88 | 0.15 | T | F | F | - |
| SIL1 | Nucleotide exchange factor SIL1 | 2.24 | -0.11 | 2.35 | 0.05 | 0.97 | 0.10 | T | F | F | - |
| SLC44A2 | Solute carrier family 44 member 2 | -1.60 | -0.60 | -1.00 | 0.04 | 0.74 | 0.26 | T | F | F | signal transduction, transport, positive regulation of I-kappaB kinase/NF-kappaB signalling, phospholipid metabolic process |
| SLFN11 | Schlafen family member 11 | -1.90 | -1.84 | -0.06 | 0.00 | 0.02 | 0.96 | T | T | F | negative regulation of G1/S transition of mitotic cell cycle, defence response to virus |
| SNAP23 | Synaptosomal-associated protein 23 | -1.65 | -0.92 | -0.73 | 0.05 | 0.57 | 0.41 | T | F | F | vesicle targeting, membrane fusion, exocytosis, membrane organization, protein transport, histamine secretion by mast cell, synaptic vesicle fusion to presynaptic membrane |
| SRI | Sorcin | -1.71 | -0.92 | -0.79 | 0.02 | 0.50 | 0.26 | T | F | F | negative regulation of transcription regulatory region DNA binding, calcium ion transport, positive regulation of insulin secretion involved in cellular response to glucose stimulus, cytoplasmic sequestering of transcription factor, proteolysis, signal transduction |
| SSB | Small RNA binding exonuclease protection factor La | 2.84 | 1.00 | 1.85 | 0.00 | 0.52 | 0.06 | T | F | F | histone mRNA metabolic process, tRNA modification. |
| STK24 | Serine/threonine kinase 24 | -1.99 | -1.59 | -0.40 | 0.03 | 0.30 | 0.69 | T | F | F | activation of protein kinase activity, execution phase of apoptosis, regulation of mitotic cell cycle, stress-activated protein kinase signalling cascade, programmed cell death, response to hydrogen peroxide, negative regulation of cell migration, protein phosphorylation |
| STXBP1 | Syntaxin-binding protein 1 | -2.55 | -1.18 | -1.37 | 0.04 | 0.63 | 0.31 | T | F | F | protein transport, vesicle docking involved in exocytosis, synaptic vesicle maturation, regulation of insulin secretion, protein stabilization, glutamate secretion, positive regulation of calcium ion-dependent exocytosis |
| SULT1A1 | Sulfotransferase family 1A member 1 | -2.68 | -2.85 | 0.17 | 0.03 | 0.11 | 0.92 | T | F | F | catecholamine metabolic process, steroid metabolic process, oestrogen metabolic process |
| TAGLN2 | Transgelin-2 | -2.18 | -2.02 | -0.16 | 0.01 | 0.10 | 0.88 | T | F | F | epithelial cell differentiation |
| THY1 | Thy-1 cell surface antigen | -2.46 | -2.16 | -0.30 | 0.04 | 0.26 | 0.83 | T | F | F | T cell receptor signalling pathway, focal adhesion assembly, negative regulation of protein kinase activity, cytoskeleton organization, negative regulation of cell migration, single organismal cell-cell adhesion |
| TNC | Tenascin C | 5.81 | 3.75 | 2.06 | 0.01 | 0.25 | 0.32 | T | F | F | cell adhesion, extracellular matrix reorganization, wound healing, positive regulation of cell proliferation. ECM proteoglycans, syndecan interactions. |
| TOM1 | Target of myb1 membrane trafficking protein | -1.61 | -0.09 | -1.52 | 0.02 | 0.97 | 0.05 | T | F | T | intracellular protein transport, endosomal transport, endocytosis |
| TPP1 | Tripeptidyl-peptidase 1 | -2.05 | 0.21 | -2.25 | 0.04 | 0.97 | 0.07 | T | F | F | endoplasmic reticulum unfolded protein response, protein catabolic process, lipid metabolic process, epithelial cell differentiation, lysosome organization, proteolysis, IRE-1-mediated unfolded protein response, peptide catabolic process, cellular protein metabolic process |
| TSG101 | Tumour susceptibility protein 101 | -1.69 | -0.33 | -1.36 | 0.05 | 0.97 | 0.18 | T | F | F | regulation of MAP kinase activity, ubiquitin-dependent protein catabolic process via the multivesicular body sorting pathway, regulation of extracellular exosome assembly, negative regulation of cell proliferation, regulation of cell growth, cellular protein modification process, cell division, membrane organization, protein transport, cell cycle arrest, endosomal transport |
| UBE2N | Ubiquitin-conjugating enzyme E2 N | -2.56 | -0.14 | -2.42 | 0.00 | 0.97 | 0.01 | T | F | T | positive regulation of DNA repair, toll-like receptor 2/4/9/5/6/10 signalling pathway, protein K63-linked ubiquitination, cytokine-mediated signalling pathway, proteolysis, innate immune response, MyD88-dependent toll-like receptor signalling pathway |
| UBR4 | Ubiquitin protein ligase E3 component N-recognin 4 | 3.15 | 0.52 | 2.63 | 0.05 | 0.97 | 0.16 | T | F | F | protein binding |
| VNN2 | Vanin 2 | -2.77 | -1.37 | -1.40 | 0.04 | 0.59 | 0.33 | T | F | F | - |
| WFDC2 | WAP four-disulfide core domain 2 | -4.45 | -3.73 | -0.72 | 0.00 | 0.02 | 0.53 | T | T | F | - |
| YTHDF2 | YTH domain-containing family protein 2 | -2.34 | -2.28 | -0.06 | 0.00 | 0.01 | 0.96 | T | T | F | - |
| YWHAH | 14-3-3 protein eta | -1.78 | -0.31 | -1.48 | 0.02 | 0.96 | 0.10 | T | F | F | programmed cell death, apoptotic process, membrane organization, gene expression regulation of sodium ion transport, glucocorticoid receptor signalling pathway, intracellular protein transport, small GTPase mediated signal transduction |

* According to ExoCarta query: http://exocarta.org/query.html
T = True: F = False.
