# Supplementary material for: Uterine Fluid Extracellular Vesicles Proteome Is Altered During the Estrous Cycle
Source: Mol Cell Proteomics. 2023 Sep 9;22(11):100642. doi: 10.1016/j.mcpro.2023.100642 (PMC10641272; doi:10.1016/j.mcpro.2023.100642)
Supplement: Supplementary file 4 [file mmc4.docx]

**Supplementary file 5: The measured uterine fluid extracellular vesicle proteins in all cows, which are previously reported to be related to endometrial receptivity in humans.** The given P-adjusted and log2 fold change values are for comparison between day 0 and 16.

| **Gene name** | **Protein name** | **P-adjusted value** | **Log2 fold change** |
| --- | --- | --- | --- |
| AGR2 | Anterior gradient 2 protein disulphate isomerase family member | 0.27 | 0.69 |
| ALPL | Alkaline phosphatase tissue-nonspecific isoenzyme | 0.01 | -3.34 |
| ANXA1 | Annexin A1 | 0.03 | -1.68 |
| ANXA2 | Annexin A2 | 0.25 | -0.69 |
| ANXA4 | Annexin A4 | 0.78 | -0.19 |
| ATP1B1 | Sodium/potassium-transporting ATPase subunit beta | 0.24 | 0.79 |
| B2M | Beta-2-microglobulin | 0.03 | -1.88 |
| CKB | Creatine kinase B-type | 0.58 | 0.27 |
| GPRC5C | G-protein-coupled receptor family C group 5 member C | 0.11 | -1.63 |
| IDH1 | Isocitrate dehydrogenase | 0.22 | -0.71 |
| MUC1 | Mucin 1 | 0.76 | 0.26 |
| MUC4 | Mucin 4 | 0.01 | -2.80 |
| MYL9 | Myosin regulatory light polypeptide 9 | 0.14 | -0.79 |
| OLFM4 | Olfactomedin 4 | 0.05 | -3.88 |
| PARK7 | Oncogene DJ1 | 0.03 | -1.33 |
| PPIA | Peptidyl-prolyl cis-trans isomerase A | 0.03 | -1.67 |
| PPIB | Peptidyl-prolyl cis-trans isomerase B | 0.35 | -0.58 |
| PRDX2 | Peroxiredoxin 2 | 0.44 | -0.37 |
| PROM1 | Prominin 1 | 0.53 | -0.66 |
| PSMB10 | Proteasome subunit beta 10 | 0.72 | -0.26 |
| RRAS | RAS related | 0.09 | -1.22 |
| S100A4 | Protein S100-A4 | 0.05 | -1.45 |
| YWHAE | 14-3-3 protein epsilon | 0.03 | -1.35 |


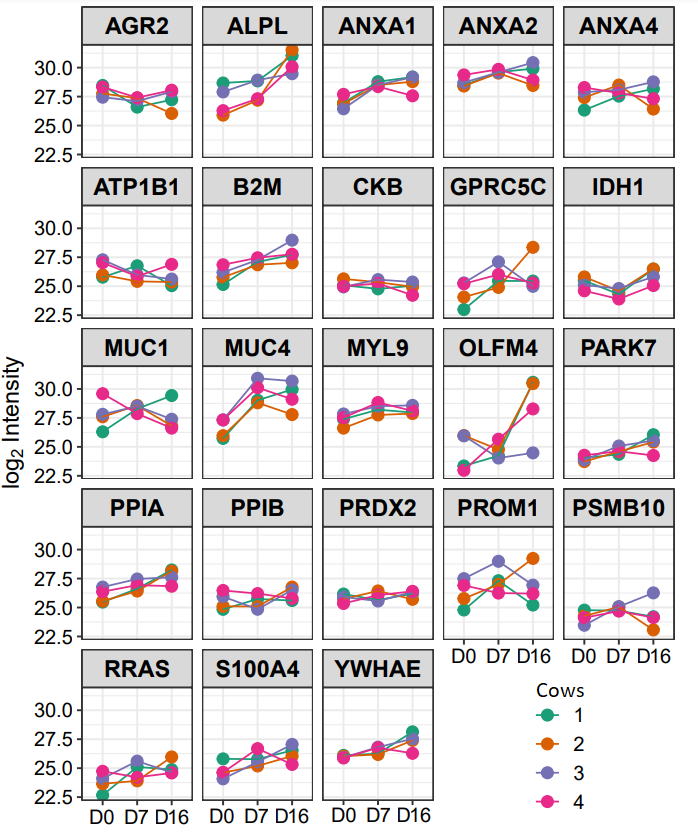


D0 = day 0 after ovulation, D7 = day 7 after ovulation, D16 = day 16 after ovulation
