# Supplementary material for: Uterine Fluid Extracellular Vesicles Proteome Is Altered During the Estrous Cycle
Source: Mol Cell Proteomics. 2023 Sep 9;22(11):100642. doi: 10.1016/j.mcpro.2023.100642 (PMC10641272; doi:10.1016/j.mcpro.2023.100642)
Supplement: Supplementary file 5 [file mmc5.docx]

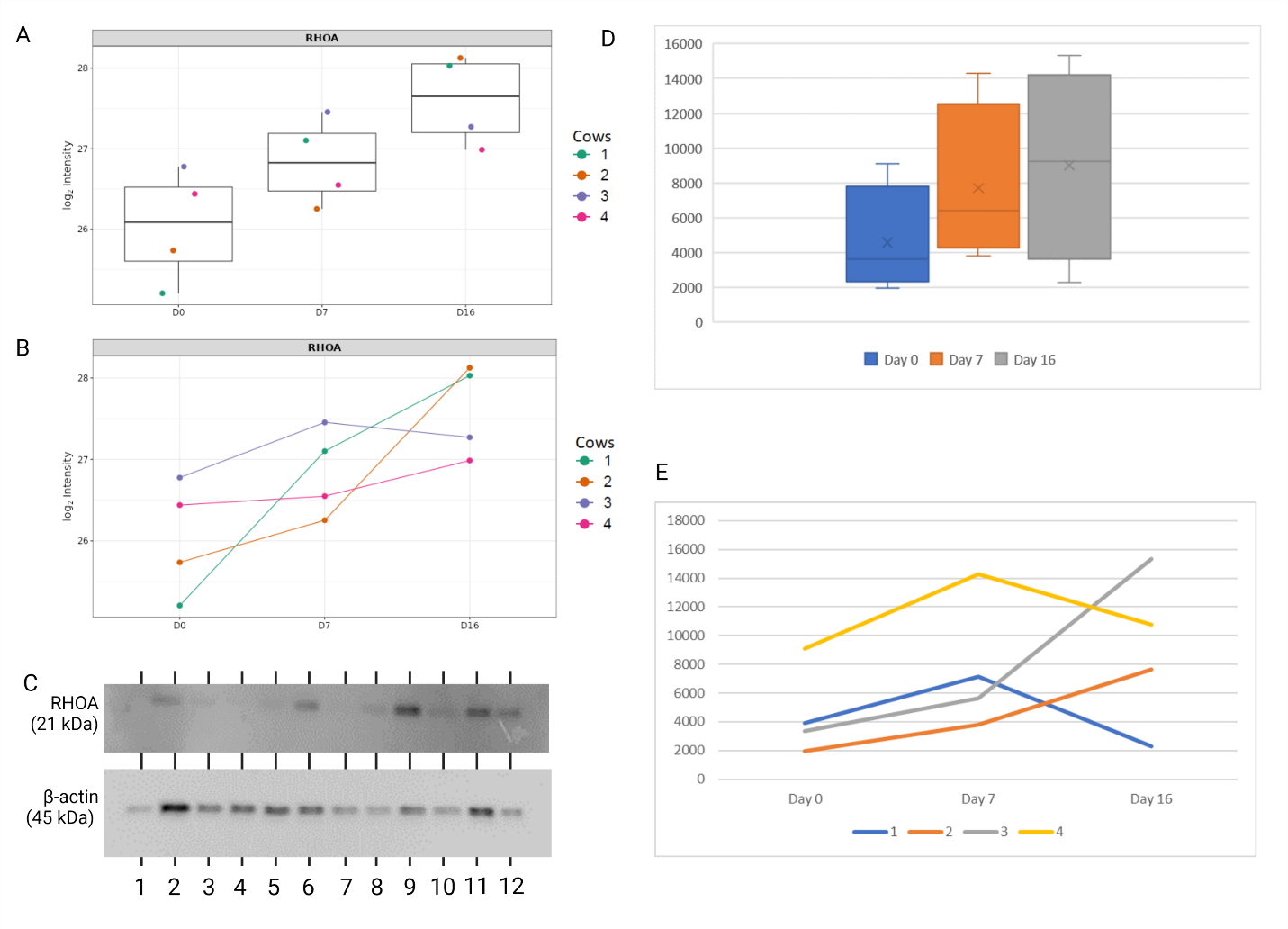
 1 – cow 1 at day 0, 2 – cow 1 at day 7, 3 – cow 1 at day 16, 4 – cow 2 at day 0, 5 – cow 2 at day 7, 6 – cow 2 at day 16, 7 – cow 3 at day 0, 8 – cow 3 at day 7, 9 – cow 3 at day 16, 10 – cow 4 at day 0, 11 – cow 4 at day 7, 12 – cow 4 at day 16 of the oestrous cycle.

**Supplementary file 6: Validation of Ras homolog family member A (RHOA) protein change measured in uterine fluid (UF) extracellular vesicles (EVs) at day 0, 7 and 16.** The liquid chromatography/mass-spectrometry-mass-spectrometry (LC-MS/MS) results showed differential higher enrichment of RHOA protein at day 16 compared to day 0 (A). However, there were variations of RHOA protein intensities between different cows when measured with LC-MS/MS (B). The LC-MS/MS results of RHOA protein was validated with Western blot analysis (WB) using UF-EV samples acquired from 2 cows (cows 3 and 4 on D-E) at day 0, 7 and 16 of the oestrous cycle, which were previously used in LC-MS/MS analysis, and 2 cow samples (cows 1 and 2 on D-E), which were not previously used in the LC-MS/MS analysis (C). The equal protein loading was evaluated using β-actin antibody (C). The increased intensity of RHOA at day 16 compared to day 0 of the oestrous cycle was confirmed using WB (D). There were variations of RHOA protein intensities between different cows (E).
