# Supplementary material for: Uterine Fluid Extracellular Vesicles Proteome Is Altered During the Estrous Cycle
Source: Mol Cell Proteomics. 2023 Sep 9;22(11):100642. doi: 10.1016/j.mcpro.2023.100642 (PMC10641272; doi:10.1016/j.mcpro.2023.100642)
Supplement: Supplementary file 6 [file mmc6.docx]

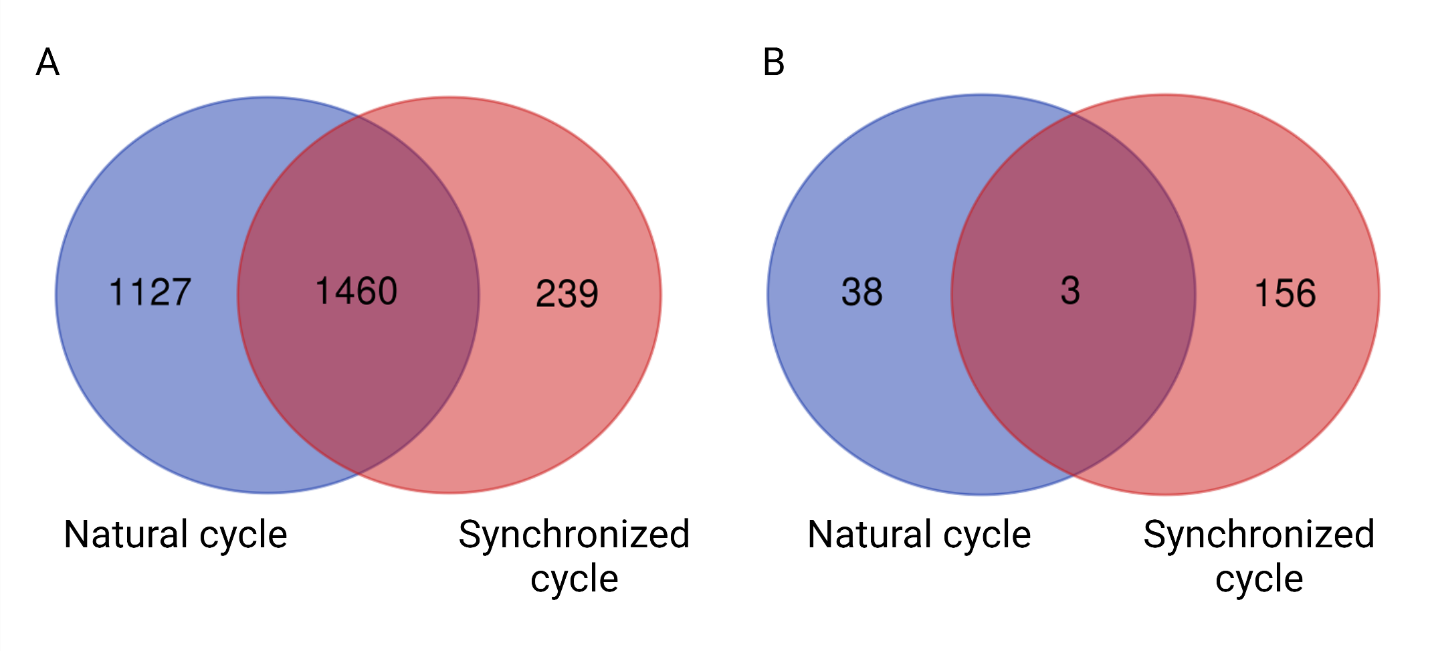


**Supplementary file 4: Overlap comparisons of bovine uterine fluid extracellular vesicle (UF-EV) related proteins between natural and synchronized cycle.** The number of identified UF-EV proteins common between natural and synchronized cycle was 1460, while 1127 UF-EV proteins were identified only in natural cycle and 239 UF-EV proteins in synchronized cycle (A). The number of differentially enriched UF-EV proteins common in natural (follicular vs luteal phase) and synchronized cycle (between days 0, 7 or 16) were 3, while 38 UF-EV proteins were differentially enriched in natural cycle and 156 UF-EV proteins in synchronized cycle.
